# Surfaceome mapping of primary human heart cells with CellSurfer uncovers cardiomyocyte surface protein LSMEM2 and proteome dynamics in failing hearts

**DOI:** 10.1038/s44161-022-00200-y

**Published:** 2023-01-16

**Authors:** Linda Berg Luecke, Matthew Waas, Jack Littrell, Melinda Wojtkiewicz, Chase Castro, Maria Burkovetskaya, Erin N. Schuette, Amanda Rae Buchberger, Jared M. Churko, Upendra Chalise, Michelle Waknitz, Shelby Konfrst, Roald Teuben, Justin Morrissette-McAlmon, Claudius Mahr, Daniel R. Anderson, Kenneth R. Boheler, Rebekah L. Gundry

**Affiliations:** 1 CardiOmics Program, Center for Heart and Vascular Research and Department of Cellular and Integrative Physiology, University of Nebraska Medical Center, Omaha, NE USA; 2 Department of Biochemistry, Medical College of Wisconsin, Milwaukee, WI USA; 3 Department of Cellular and Molecular Medicine and Sarver Molecular Cardiovascular Research Program, University of Arizona, Tucson, AZ USA; 4 Department of Biomedical Engineering, Whiting School of Engineering, The Johns Hopkins University, Baltimore, MD USA; 5 Department of Mechanical Engineering, Division of Cardiology, University of Washington, Seattle, WA USA; 6 Division of Cardiovascular Medicine, University of Nebraska Medical Center, Omaha, NE USA; 7 Department of Medicine, Division of Cardiology, The Johns Hopkins University, Baltimore, MD USA; 8 Present Address: Princess Margaret Cancer Centre, University Health Network, Toronto, ON Canada; 9 Present Address: Department of Chemistry, University of Wisconsin-Madison, Madison, WI USA

**Keywords:** Proteomics, Stem-cell differentiation, Systems analysis, Mass spectrometry, Glycosylation

## Abstract

Cardiac cell surface proteins are drug targets and useful biomarkers for discriminating among cellular phenotypes and disease states. Here we developed an analytical platform, CellSurfer, that enables quantitative cell surface proteome (surfaceome) profiling of cells present in limited quantities, and we apply it to isolated primary human heart cells. We report experimental evidence of surface localization and extracellular domains for 1,144 *N-*glycoproteins, including cell-type-restricted and region-restricted glycoproteins. We identified a surface protein specific for healthy cardiomyocytes, LSMEM2, and validated an anti-LSMEM2 monoclonal antibody for flow cytometry and imaging. Surfaceome comparisons among pluripotent stem cell derivatives and their primary counterparts highlighted important differences with direct implications for drug screening and disease modeling. Finally, 20% of cell surface proteins, including LSMEM2, were differentially abundant between failing and non-failing cardiomyocytes. These results represent a rich resource to advance development of cell type and organ-specific targets for drug delivery, disease modeling, immunophenotyping and in vivo imaging

## Abstract (web summary)

Berg Luecke et al. developed an analytical platform, CellSurfer, that enables the quantitative profiling of cell surface proteome (surfaceome) from small samples, and they apply it to primary human heart cells. The analyses revealed LSMEM2 as a surface protein specific for healthy cardiomyocytes; important surfaceome differences between primary human cardiac cells and the pluripotent stem cell derivatives; and differences in the abundance of surface proteins between human failing and non-failing cardiomyocytes.

## Main

Cardiomyocytes, fibroblasts, endothelial cells, smooth muscle cells and other heart cells work together to keep blood flowing throughout the body. Central to these coordinated heart functions are cell surface proteins (surfaceome^1^) that detect and transmit signals between cells. The surfaceome differs among cell types, and proteins on the cell surface change in response to internal and external signals activated during physiological development or pathological conditions. These attributes, together with their extracellular epitopes, make cell surface proteins suitable targets for reagents used in live cell sorting and in vivo imaging^2,3^. Clinically, cell surface proteins are a rich source for biomarkers that distinguish cellular phenotypes and disease states and for targets of drugs or compounds useful in the treatment of cardiac disorders or diseases^4^.

Despite previous molecular profiling efforts^5–8^, a detailed cell type and spatially resolved view of the surfaceome of the adult human heart is lacking. Transcriptomic approaches cannot directly inform protein abundance or localization. Antibody-based strategies are limited by availability of high-quality reagents, and generic proteomic analyses undersample the plasma membrane and do not directly inform subcellular localization. Consequently, there is an incomplete understanding of proteins and signaling pathways differentially present in normal or diseased myocardium, a limited number of accessible targets for organ-directed and cell-type-directed drug delivery and few validated cell-type-specific reagents for cell sorting and imaging of cardiac cells.

To enable surfaceome profiling of primary human cardiac cells, we developed an analytical platform, CellSurfer, which overcomes previous challenges in mass spectrometry (MS)-based cell surface proteome studies. CellSurfer includes a microscale Cell Surface Capture (µCSC) method for discovery of cell surface *N-*glycoproteins from small sample sizes as well as Veneer, a bioinformatic tool that expedites analysis, annotation and candidate prioritization for downstream validation. We applied CellSurfer to generate experimentally derived surfaceome maps of primary isolated human cardiac cells. These results advance the use of cell-type-specific and region-specific surfaceome data for improved drug evaluations, better disease modeling and testing biomarkers for heart disease.

## Results

### CellSurfer platform

**Fig. 1:**
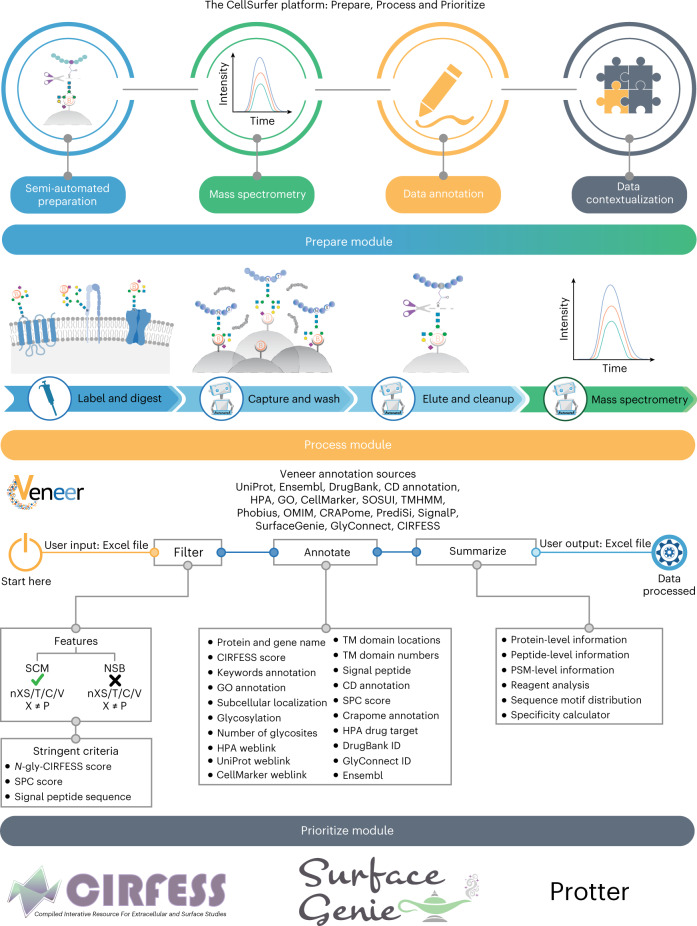
Overview of the CellSurfer platform. The Prepare module combines the μCSC method for sample preparation with MS for identification and quantification of extracellular domains of cell surface *N-*glycoproteins. The Process module features Veneer, a web-based bioinformatic tool to rapidly curate search engine results, provide enhanced functional annotations and enable consistent reporting and distribution of CSC and related datasets. The Prioritize module offers the incorporation of multiple tools to prioritize candidate markers and assess and visualize surfaceome coverage.

CellSurfer comprises three modules within a streamlined platform for rapid, quantitative discovery of the cell surface *N-*glycoproteome (Fig. 1). The ‘Prepare module’ features a μCSC method for sample preparation, identification and quantification of extracellular domains of cell surface *N-*glycoproteins by MS. μCSC uses a chemical catalyst to enhance hydrazide-mediated biotinylation of extracellular glycans on cells with intact plasma membranes and an automated magnetic-bead-based glycopeptide enrichment strategy to minimize sample loss and human intervention. The ‘Process module’ features Veneer, a webtool that rapidly curates search engine results and provides enhanced functional annotations to facilitate biological interpretation (https://www.cellsurfer.net/veneer). Veneer enables consistent interpretation and reporting of μCSC and related datasets. We avoid reliance on Gene Ontology (GO) terms and database annotations—commonly used in transcriptomic and generic proteomics approaches—by using experimental evidence and applying stringent criteria to accurately classify identified proteins as cell surface *N-*glycoproteins. The *N-*glycosite information is valuable for determining the orientation of uncharacterized transmembrane proteins necessary to inform downstream antibody development. In the ‘Prioritize module’, CIRFESS^9^ provides a quality control measure to match predicted versus experimentally identified *N*-glycosylated extracellular peptides. SurfaceGenie^10^ rapidly prioritizes candidate cell-type-specific markers. Protter^11^ provides visualization of experimentally identified peptides as it relates to membrane protein topology, providing a rapid strategy to view the extracellular domains of proteins and inform epitope selection for antibody or other targeting. Combining these tools allows users to rapidly prioritize candidate markers and assess and visualize surfaceome coverage and extracellular domains for downstream applications (for example, drug targeting, immunophenotyping and immunotherapy).

### µCSC enables surfaceome profiling from small sample sizes

**Extended Data Fig. 1:**
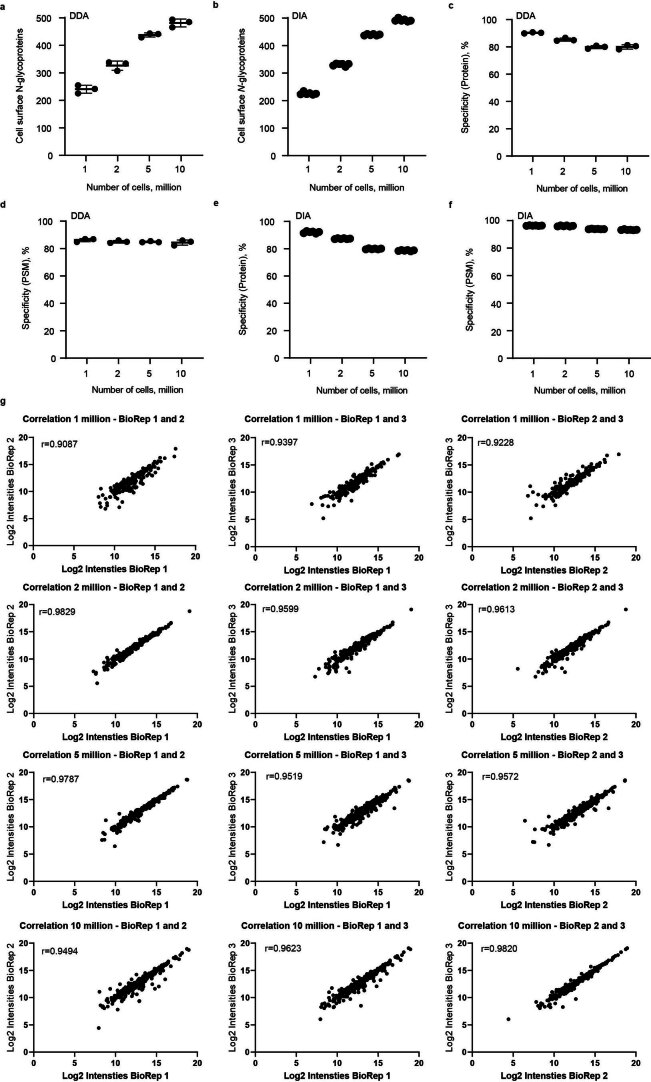
Overview of CellSurfer analysis of B cells. **a**. Total number of cell surface *N*-glycoproteins identified in µCSC experiments for 1–10 million B cells (RPMI 1788) acquired in DDA mode and **b**. DIA mode. **c**. Protein level specificity calculated based on the total number of cell surface *N*-glycoproteins divided by total number of proteins identified in DDA mode and **d**. Peptide spectrum match (PSM) level specificity calculated based on the total number of PSM with a deamidation within the sequence consensus motif divided by total number of PSM identified in DDA mode. **e**. Protein and **f**. PSM level specificity for experiments acquired in DIA mode. **g**. Assessment of reproducibility of µCSC performed on B cells (RPMI 1788) acquired in DIA mode. Scatter plot visualizing log_2_ intensities of biological replicates (BioRep) and Pearson correlation coefficient for 1–10 million cells acquired in DIA mode. All panels: Scatter plots are shown with mean and s.d. and are 3 distinct biological replicates, 2 technical injections/replicates per biological replicate. Source data

**Extended Data Fig. 2:**
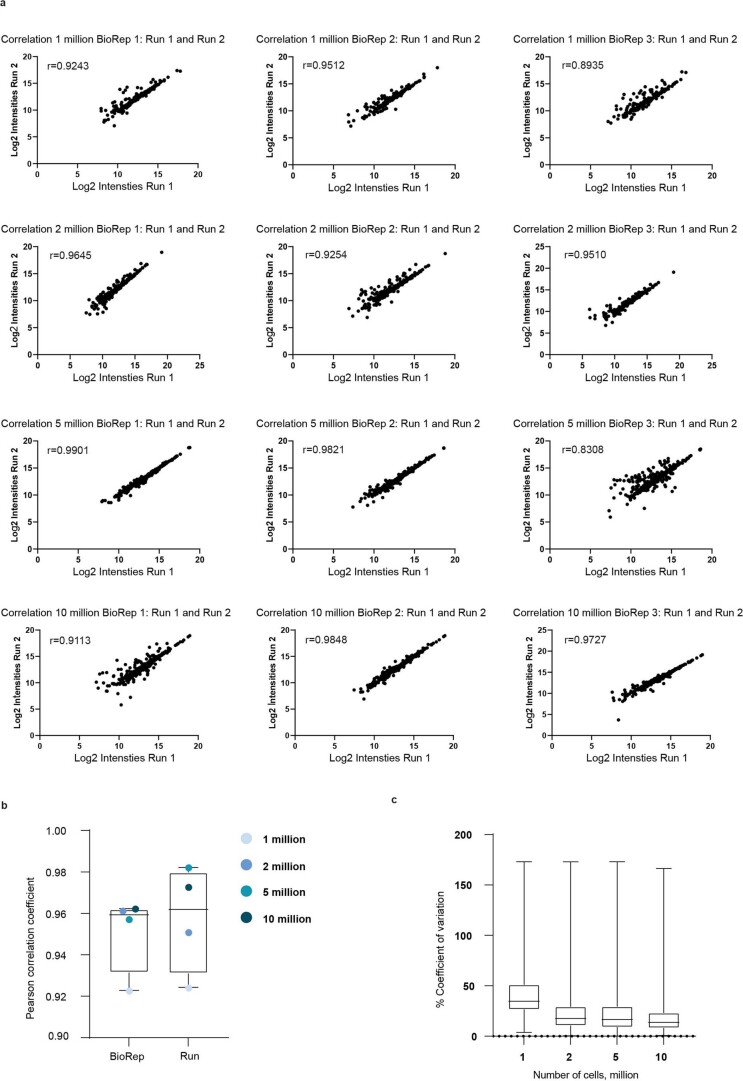
Assessment of reproducibility of µCSC performed on B cells (RPMI 1788) acquired in DIA mode. **a**. Scatter plot visualizing log_2_ intensities of runs per biological replicate (BioRep) and Pearson correlation coefficient for 1–10 million cells. **b**. Boxplot highlighting median Pearson correlation coefficient of BioRep and run for 1–10 million cells. Median with interquartile ranges shown (upper and lower) and whiskers show min to max. **c**. Boxplot showing percent coefficient of variation of *N*-glycopeptide raw intensities for 1–10 million cells. Median with interquartile ranges shown (upper and lower) and whiskers show min to max. All panels: 3 distinct biological replicates, 2 technical injections/replicates per biological replicate. Source data

We validated µCSC performance using human B cells, which have been well characterized in previous surfaceome studies^12–14^. Starting with 1, 2, 5 and 10 million B cells, we identified 276, 351, 470 and 501 *N*-glycoproteins, respectively, using data-dependent acquisition (DDA). Data-independent acquisition (DIA) performed similarly (Extended Data Figs. 1 and 2). Capture specificity for high-confidence cell surface proteins was more than 80% at the protein and peptide spectrum match (PSM) levels for all experiments. Protein abundance was consistent among biological and technical replicates, with median Pearson correlation coefficients of 0.959 and 0.962, respectively. Median percent coefficients of variation for raw intensities of *N*-glycopeptides were 34.8%, 17.6%, 16.6% and 13.7% for 1, 2, 5 and 10 million cells, respectively.

**Extended Data Fig. 3:**
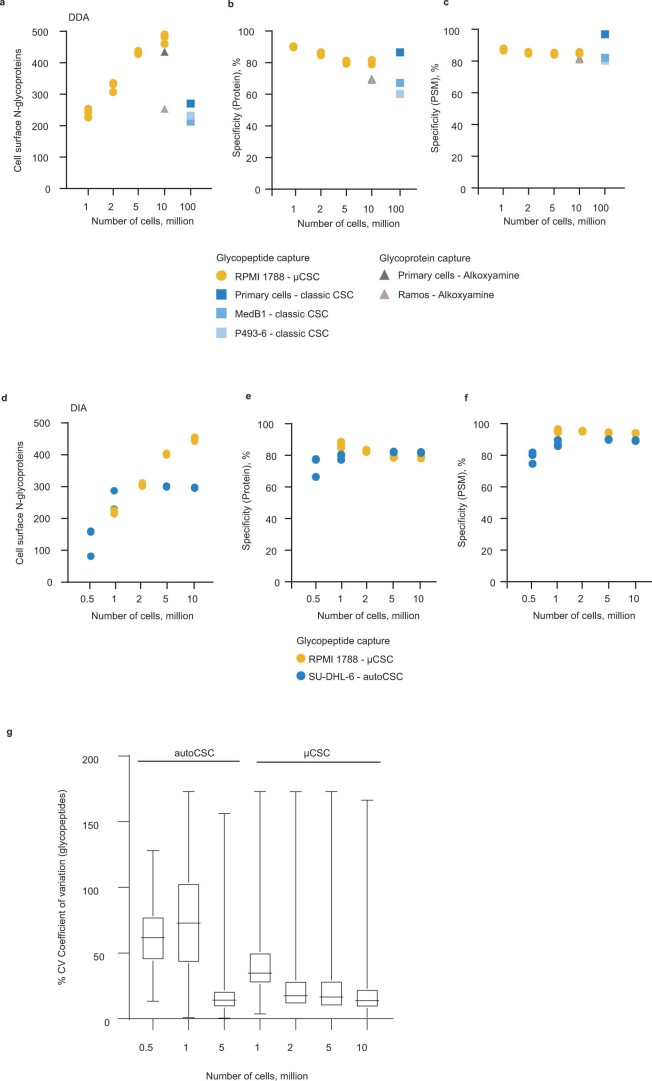
Comparison of µCSC to previously published datasets. **a**. Total number cell surface *N*-glycoproteins identified in µCSC experiments for 1–10 million RPMI 1788 cells in comparison to previously published B cell datasets by Kalxdorf et al. (primary B cells and Ramos)^13^ and Bausch-Fluck et al. (primary B cells, MedB1, and P493-6)^12^ acquired in DDA mode. **b**. Protein and **c**. PSM level specificity. Each circle, triangle, or square represents 1 biological replicate. **d**. Total number cell surface *N*-glycoproteins identified in µCSC experiments for 1–10 million RPMI 1788 cells in comparison to previously published B cell dataset by van Oostrum et al. (SU-DHL-6)^14^ acquired in DIA mode. **e**. Protein and **f**. PSM level specificity. Each blue circle represents 1 biological replicate. Each yellow circle represents 1 technical replicate. Three distinct biological replicates were used for RPMI 1788. **g**. Comparison of µCSC to autoCSC^20^. 3 distinct biological replicates, 2 technical injections/replicates per biological replicate. Boxplot showing percent coefficient of variation of *N*-glycopeptide raw intensities for 0.5–10 million cells. Median with interquartile ranges shown (upper and lower) and whiskers show min to max. Source data

In comparison to previous surfaceome methods, the number of proteins identified by μCSC from 1 million cells is similar to classic CSC starting with 100 million cells^12^ (Extended Data Fig. 3). Specificity for *N-*glycoproteins is higher for μCSC than reported by alkoxyamine-PEG_4_-biotinylation approaches and on par with classic CSC. In comparison to autoCSC^14^, the μCSC method identifies similar numbers of proteins for 1 million cells but more proteins for 5 million and 10 million cells. Percent coefficients of variation for raw intensities of *N*-glycopeptides of μCSC were lower for smaller numbers of cells compared to autoCSC (Extended Data Fig. 3). Whereas autoCSC requires pipette tips commercially prepared by custom order^14^, μCSC uses only broadly available commercial reagents and is compatible with any liquid handling workstation capable of magnetic-bead-based separations. Altogether, μCSC is specific, repeatable and well suited to the qualitative and quantitative analysis of small numbers of cells.

### Surfaceome maps of primary human adult cardiac cells

**Fig. 2:**
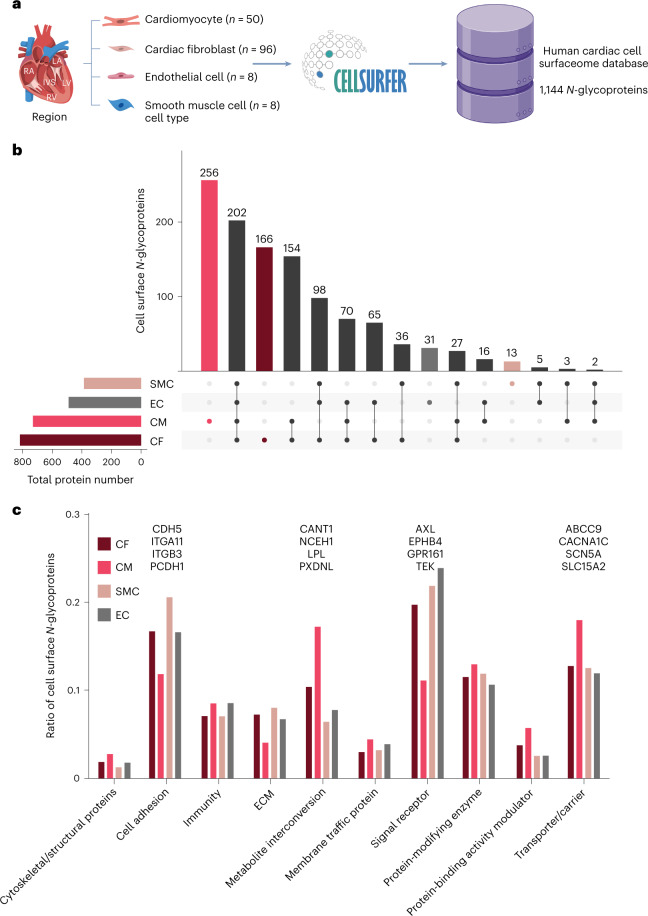
Human cardiac cell surface *N-*glycoproteome. **a**, Schematic overview for generating a reference catalog of cell surface *N*-glycoproteins of primary human cardiac cells. The number of μCSC experiments for each cell type is indicated. **b**, UpSet plot^76^ visualizing the intersections of identified proteins from human primary cardiomyocytes (CMs), cardiac fibroblasts (CFs), cardiac microvascular endothelial cells (ECs) and coronary artery smooth muscle cells (SMCs) that includes DDA and DIA data. **c**, Bar graph summarizing the protein classes common among CMs, CFs, ECs and SMCs. Protein classes were assigned based on GO term analysis. Ratios were determined by dividing the number of cell surface *N*-glycoproteins in a specific protein class by the total number of cell surface *N*-glycoproteins for that cell type. Examples of proteins identified in CFs, ECs and SMCs for cell adhesion (CDH5, ITGA11, ITGB3 and PCDH1) and signal receptor (AXL, EPHB4, GPR161 and TEK) are indicated by gene name. Examples of proteins identified exclusively in CMs for metabolite interconversion (CANT1, NCEH1, LPL and PXDNL) and transport/carrier (ABCC9, CACNA1C, SCN5A and SLC15A2) are indicated by gene name. Parts of this figure were generated with BioRender.

**Extended Data Fig. 4:**
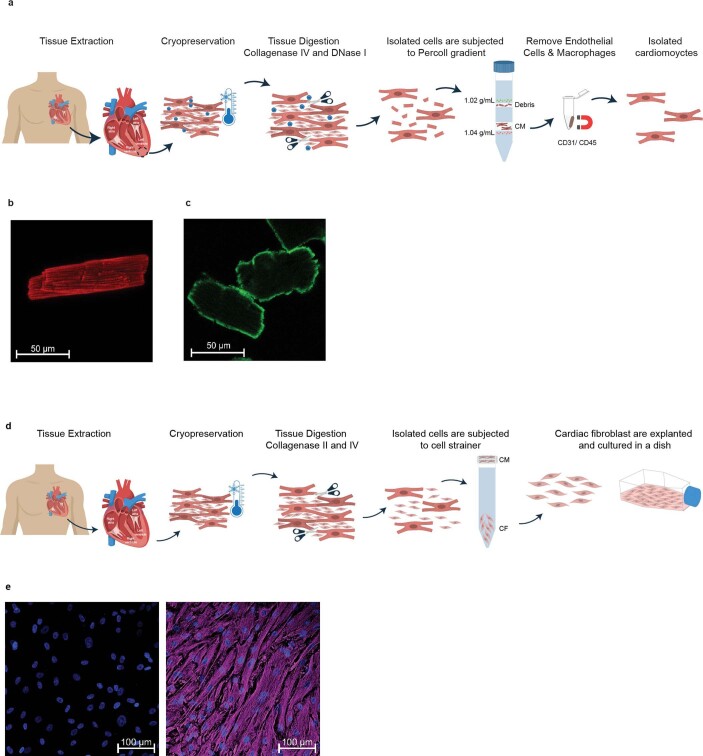
Isolation and assessment of cardiomyocytes. **a**. Schematic overview of cardiomyocyte isolation workflow using cryopreserved human adult cardiac tissue. **b**. Representative confocal immunofluorescence images of isolated cardiomyocytes stained for cardiac actin (red; 7 biological replicates) and **c**. streptavidin (green; 5 biological replicates). **d**. Isolation and assessment of cardiac fibroblasts. Schematic overview of cardiac fibroblasts isolation workflow using cryopreserved human adult cardiac tissue. **e**. Representative confocal immunofluorescence images of isolated and cultured cardiac fibroblasts stained for vimentin (purple; 3 biological replicates) and nuclei (blue) at Passage 0. Scale bar, 100 µm. Parts of this Fig. were generated with BioRender.com.

We used the CellSurfer platform to develop a reference surfaceome of isolated, primary human adult cardiac cells (Fig. 2a and Extended Data Fig. 4). We identified 1,144 cell surface *N-*glycoproteins across four cell types, including 730, 817, 489 and 386 on cardiomyocytes, cardiac fibroblasts, cardiac microvascular endothelial cells and coronary artery smooth muscle cells, respectively (Supplementary Table 1). These data include 150 cluster of differentiation (CD) molecules, 171 transporters and channels and 238 receptors, including 71 G-protein-coupled receptors. Of 1,144 proteins, 73 lacked previous protein-level evidence per UniProt annotations, and 265 are known drug targets, of which 77 drugs are indicated for cardiac diseases (DrugBank).

In total, 466 proteins were observed exclusively on a single cell type, including 256, 166, 31 and 13 on cardiomyocytes, cardiac fibroblasts, cardiac microvascular endothelial cells and coronary artery smooth muscle cells, respectively (Fig. 2b). Relative to these other cells, cardiomyocytes have increased ratios of glycoproteins involved in metabolite interconversion and molecule transport, including glycoproteins exclusively identified in cardiomyocytes (for example, ABCC9, CACNA1C and LPL; Fig. 2c). Comparatively, cardiac fibroblasts, microvascular endothelial cells and coronary artery smooth muscle cells collectively had increased ratios of glycoproteins involved in cell adhesion (for example, CDH5 and ITGA11) and signaling (for example, AXL and GPR161). These results are expected as the contractile function of cardiomyocytes requires an extensive ion channel network to propagate action potentials, whereas cell adhesion proteins are known to mediate pro-inflammatory processes in cardiac fibroblasts, endothelial cells and smooth muscle cells.

### The cardiac cell surfaceome informs research applications

**Fig. 3:**
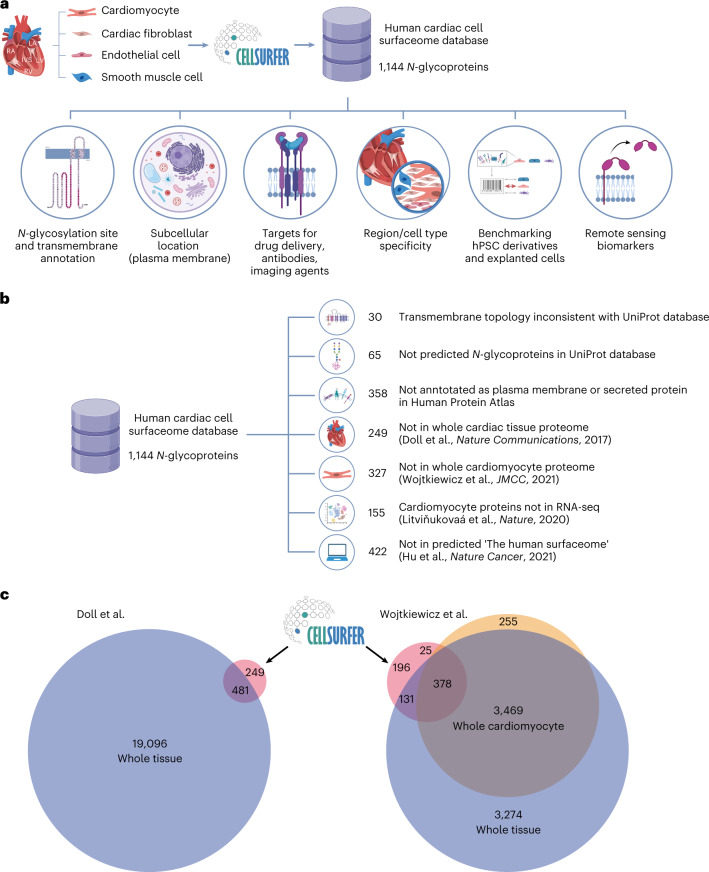
Versatility of CellSurfer to inform a broad range of applications. Examples are provided for cardiac cell data, but concepts are applicable to any cell type. **a**, CellSurfer enables the discovery of organ-specific, cell-type-specific and region-specific cell surface *N*-glycoproteins from small numbers of human primary cells. The resulting data inform the orientation of transmembrane proteins and *N-*glycosites for proteins localized to the cell surface—information that is essential for informing drug and antibody development. Proteins can be further interrogated as possible targets for drug and antibody development and benchmarking stem cell derivatives and explanted cells. Secreted and shed proteins can serve as possible remote sensing markers. **b**, Cardiac cell surfaceome highlights inconsistencies with database annotations and limitations of prediction and transcriptomic technologies. **c**, Venn diagram shows comparison of the cardiac cell surfaceome with proteins identified by generic whole cardiac tissue proteomics (Doll et al.^5^) and by generic whole cardiac tissue proteomics and cardiomyocyte proteomics performed by our laboratory^16^. The purple, orange and pink circles represent the number of proteins identified in whole tissue, whole cardiomyocytes and cardiomyocytes processed by CellSurfer, respectively. Parts of this figure were generated with BioRender. RNA-seq, RNA sequencing.

CellSurfer data reveal *N-*glycosylated peptides exposed in the extracellular domain, and Veneer is designed to provide easy interpretation of *N-*glycosite information valuable for determining the orientation of transmembrane proteins (Supplementary Tables 1 and 2). CellSurfer data are valuable for a range of research applications, including updating public repositories regarding *N-*glycosylation sites, topology predictions for transmembrane proteins and subcellular localizations in a cell-type-specific manner (Fig. 3a). This information is essential for informing drug and antibody development and solving bioinformatic prediction discrepancies. CellSurfer results viewed in Protter reveal that experimental evidence of transmembrane protein orientation for 30 proteins are inconsistent with entries in the current version of UniProt (Fig. 3b and Supplementary Table 3). Sixty-five *N-*glycoproteins identified here are not predicted *N-*glycoproteins in UniProt, including HTR6, LSMEM2 and ABCB6 (Fig. 3b and Supplementary Table 4). Of the 1,144 cell surface *N-*glycoproteins identified in human cardiac cells, hundreds are not annotated as either plasma membrane or secreted, or are not documented as being present in the heart based on immunohistochemistry data in the Human Protein Atlas (HPA; Fig. 3b and Supplementary Table 5). Given the discrepancies with antibody-based localization assays, this cardiac cell surfaceome will be instrumental for updating public repositories regarding cell-type-specific protein localization, *N-*glycosylation sites and topology predictions for transmembrane proteins.

Application of CellSurfer to identify and characterize the surfaceome of isolated cells has advantages over transcriptomics, computational approaches and generic proteomics. Twenty-one percent of the cardiomyocyte cell surface *N-*glycoproteins were not identified by RNA sequencing (single-nucleus and single-cell RNA sequencing) in a study by Litviňukovaá et al.^6^ (Fig. 3b). Thirty-eight percent (442) of experimentally determined surface *N-*glycoproteins were not included in the 3,567 proteins defined as the human surfaceome based on nine experimental and computational resources defined by Hu et al.^15^. Of the 730 cardiomyocyte surface *N-*glycoproteins identified, 249 were not identified among the nearly 20,000 proteins identified by whole cardiac tissue proteomics performed by Doll et al.^5^ (Fig. 3c). In our study, 68 of these were uniquely identified on cardiomyocytes, emphasizing the importance of cell-type-specific approaches for mapping the surfaceome. Similarly, 221 and 327 proteins were not identified by whole cardiac tissue and whole isolated cardiomyocyte proteomics performed by our laboratory^16^, respectively (Fig. 3c). Although these studies identified proteins observed by CellSurfer, the proteomic approaches used previously do not provide evidence of subcellular location, and whole tissue analyses do not provide evidence of cell type.

### LSMEM2 is a cardiomyocyte-restricted cell surface protein

**Fig. 4:**
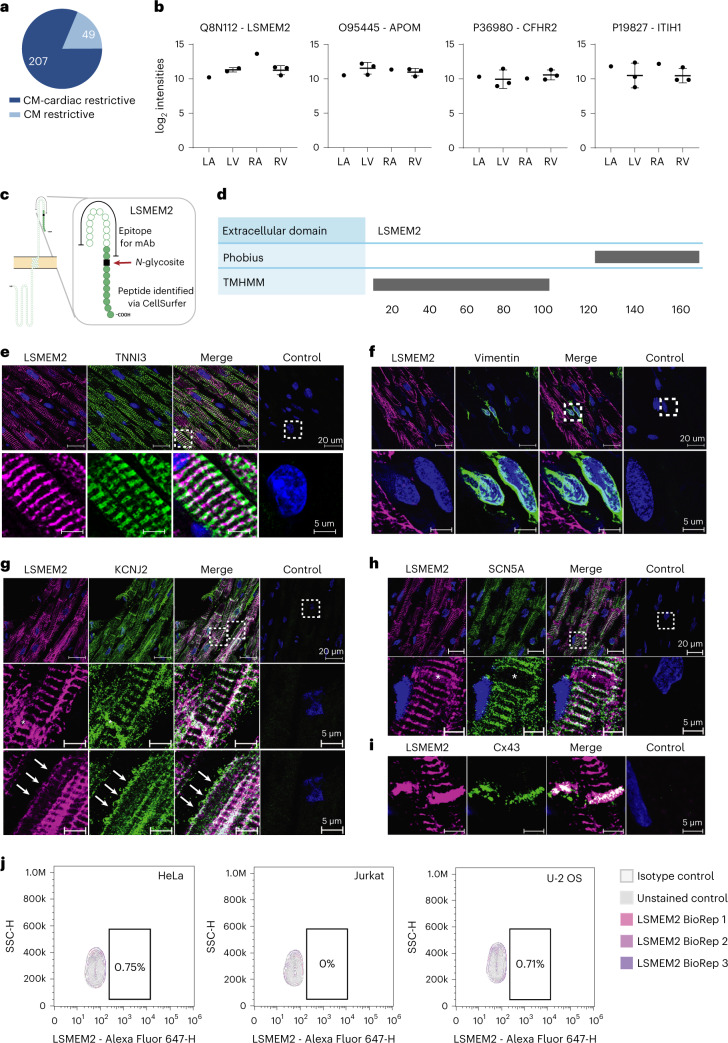
LSMEM2 is a previously uncharacterized protein on the surface of cardiomyocytes. **a**, Pie chart visualizing the number of proteins restrictive to cardiomyocytes within the heart or restrictive to cardiomyocytes when compared to 68 other non-cardiac cell types. **b**, log_2_ intensity values for LSMEM2, APOM, CFHR2 and ITIH1 for pooled LA and RA samples (six pooled donors for each LA and RA) and three matched donors for LV and RV. Scatter plots are shown with mean and s.d. Proteins are reported by UniProt accession and gene name. **c**, Protter^11^ image of LSMEM2 visualizing the epitope used to develop an mAb against LSMEM2 and the *N-*glycopeptide identified by CellSurfer. **d**, CIRFESS^9^ output showing the conflicting prediction of the extracellular domain of LSMEM2 by Phobius and TMHMM. **e**, Representative confocal immunofluorescence images of human cardiac tissue stained for LSMEM2 (purple), cardiac troponin I (TNNI3, green) and nuclei (blue). **f**, Representative confocal immunofluorescence images of human cardiac tissue stained for LSMEM2 (purple), vimentin (green) and nuclei (blue). Scale bars, 20 µm (top panel) and 5 µm (bottom panel). **g**, Representative confocal immunofluorescence images of cardiac tissue stained for LSMEM2 (purple), KCNJ2 (green) and nuclei (blue). Intercalated discs are marked by asterisks. Cell membrane is marked by arrows. Scale bars, 20 µm (top panel) and 5 µm (middle and bottom panel). **h**, Representative confocal immunofluorescence images of cardiac tissue stained for LSMEM2 (purple), SCN5A (green) and nuclei (blue). Intercalated discs are marked by asterisks. Scale bars, 20 µm (top panel) and 5 µm (bottom panel). **i**, Representative confocal immunofluorescence images of cardiac tissue stained for LSMEM2 (purple), connexin 43 (Cx43, green) and nuclei (blue). Scale bar, 5 µm. **j**, Flow cytometry contour plots depicting live cell flow cytometry of LSMEM2 on HeLa, Jurkat and U-2 OS cells. **e**–**i**, 10, 3, 3, 2 and 6 biological replicates for LSMEM2, KCNJ2, SCN5A, vimentin and CX43, respectively. **j**, Three biological replicates for each cell line. Source data

**Extended Data Fig. 5:**
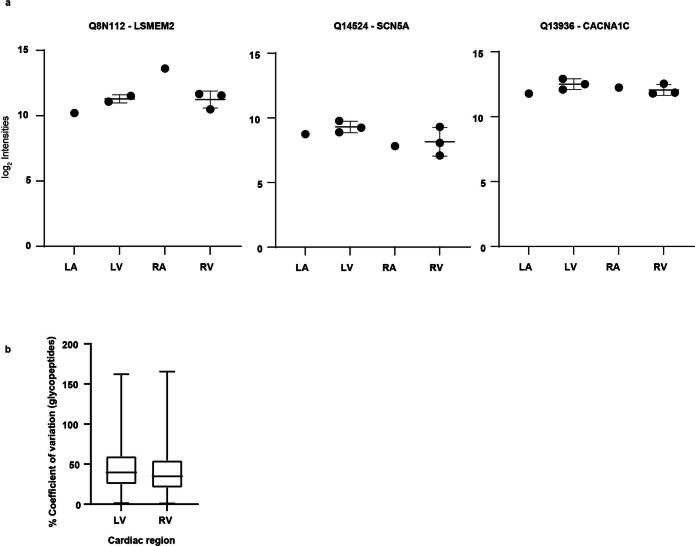
DIA analysis of surface proteins among heart chambers. **a**. log_2_ intensity values from DIA analysis of LSMEM2, SCN5A, and CACN1AC for pooled left and right atria (LA and RA) samples (6 pooled donors for each LA and RA) and 3 matched donors for left and right ventricles (LV and RV). Proteins are both reported by UniProt accession and gene name. Scatter plots are shown with mean and s.d.. **b**. Boxplot showing percent coefficient of variation of *N*-glycopeptide raw intensities for cardiomyocytes isolated from left and right ventricle (LV and RV). Median with interquartile ranges shown (upper and lower) and whiskers show min to max. 3 biological replicates each for LV and RV. Source data

Leucine-rich single-pass membrane protein 2 (LSMEM2) is an example of the importance of experimental evidence when investigating previously uncharacterized cell surface proteins. To identify potential cell-type-restricted markers, we compared 256 *N-*glycoproteins identified uniquely in cardiomyocytes to more than 2,500 cell surface *N-*glycoproteins observed across 68 non-cardiac human cell types, including 27 in the Cell Surface Protein Atlas^12^ (Supplementary Table 6). These comparisons reveal 49 proteins uniquely identified in cardiomyocytes, of which 14 were identified in cardiomyocytes isolated from four or five regions of the heart (Fig. 4a). DIA MS of these 14 proteins reveals that 13, including LSMEM2, APOM, CFHR2 and ITIH1 (Fig. 4b, Supplementary Table 7), were similar in abundance across cardiomyocytes isolated from all cardiac regions, as were known proteins SCN5A and CACNA1C (Extended Data Fig. 5a).

We identified LSMEM2 as a high-priority cardiomyocyte-restricted candidate because we have only observed it previously on the surface of human pluripotent stem-cell-derived cardiomyocytes (hPSC-CMs)^17^^,18^. It was inferred from the detection of one extracellular glycopeptide unique to LSMEM2 among the human proteome^9^ (Fig. 4c). LSMEM2 was a predicted single-pass but uncharacterized transmembrane protein. Its membrane orientation was unclear because Phobius^19^ and TMHMM^20^ algorithms yield conflicting predictions (Fig. 4c,d). UniProt did not predict LSMEM2 to contain an *N*-glycosite, possibly because of discrepant transmembrane predictions. Finally, LSMEM2 was not annotated as a plasma membrane protein in previous releases (for example, version 13 and version 14) of the HPA^21^, but, in the current version (version 21), LSMEM2 is annotated as a plasma membrane protein.

**Extended Data Fig. 6:**
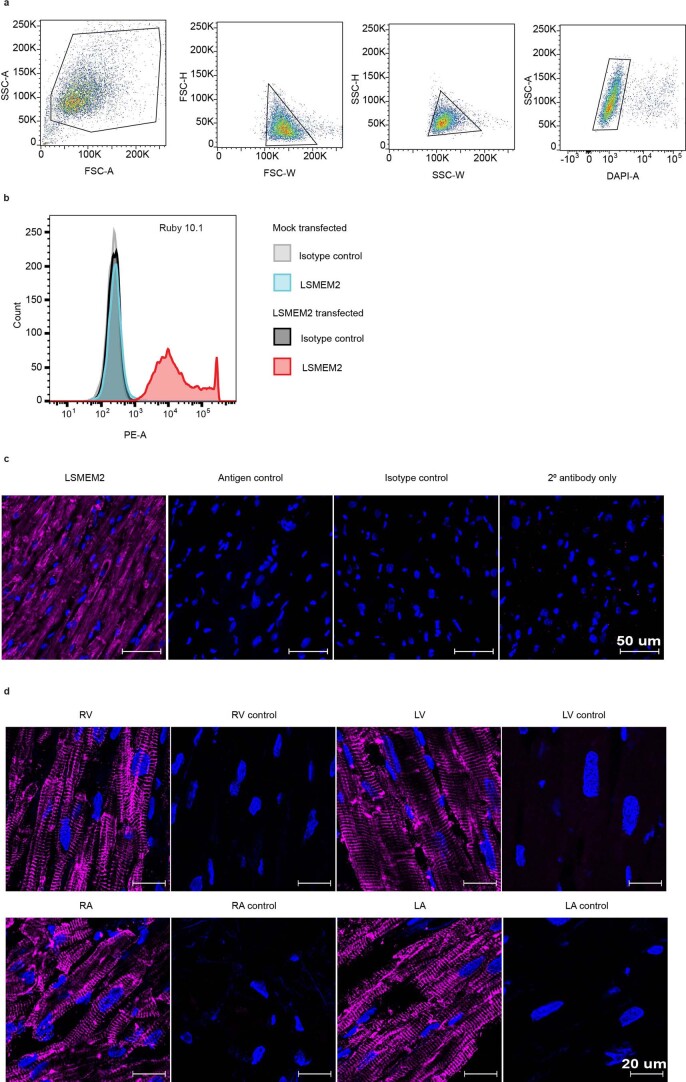
Monoclonal antibody validation of Ruby 10.1. **a**. Gating strategy applied to 293T live-cell flow cytometry experiments. **b**. Histogram of isolated 293T cells for Ruby 10.1. Representative of 3 biological experiments. **c-d**. Imaging of LSMEM2 in human cardiac tissue. **d**. Representative confocal immunofluorescence images of cardiac tissue stained for LSMEM2 (purple), nuclei (blue), and the following controls: antigen control (blocking peptides), isotype control, and secondary antibody only control. Scale bar, 50 µm. 3 biological replicates. **d**. Representative confocal immunofluorescence images of cardiac tissue from four chambers left and right ventricle (LV and RV) and left and right atria (LA and RA) stained for LSMEM2 (purple) and nuclei (blue). Scale bar, 20 µm. Representative of 3 biological experiments for LV, RV, RA; 2 biological replicates for LA.

Surprisingly, HPA immunocytochemistry data suggest that the polyclonal anti-LSMEM2 antibody HPA079523 binds to the surface of HeLa, Jurkat T and U-2 OS cells. These results are inconsistent with published MS-based surfaceome analyses that did not detect LSMEM2 protein in any of these cell types^12,14^. As no monoclonal antibodies (mAbs) for LSMEM2 were available, we generated an mAb (clone Ruby10.1) based on the extracellular epitope identified for LSMEM2 (Fig. 4c). Specificity of Ruby10.1 was validated by flow cytometry using mock-transfected and LSMEM2-transfected 293T cells (Extended Data Fig. 6a,b). Immunohistochemistry of human cardiac tissue demonstrates that Ruby10.1 is specific (Extended Data Fig. 6c), localizes only to troponin-positive cells (cardiomyocytes of all four regions; Extended Data Fig. 6d and Fig. 4e) and was not detected in vimentin-positive cells (cardiac fibroblasts and endothelial cells; Fig. 4f). In cardiomyocytes, the Ruby10.1 immunodetection pattern is similar, but not identical, to that of ion channels KCNJ2 and SCN5A (Fig. 4g,h) and localizes to intercalated discs (Fig. 4i).

**Extended Data Fig. 7:**
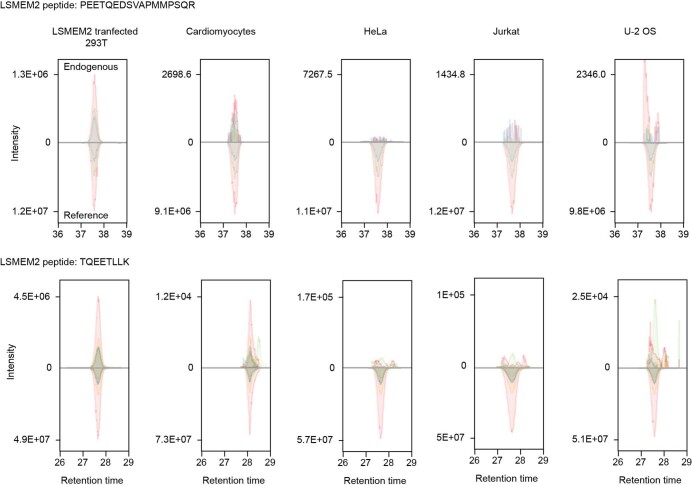
Targeted MS analysis of two LSMEM2 peptides across five cell types depicted in extracted ion chromatograms. Both endogenous LSMEM2 peptides are detected with appropriate overlap with the isotopically labeled internal standards (reference signal is lower panel) only in 293T cells overexpressing LSMEM2 and primary cardiomyocytes, and are not detected in the HeLa, Jurkat, and U2-OS cells. 3 biological replicates.

In contrast to the HPA, our analyses of HeLa, Jurkat T and U-2 OS cells did not detect LSMEM2 with Ruby10.1 mAb by flow cytometry (Fig. 4j). Targeted MS analyses of two non-glycosylated LSMEM2 peptides provide orthogonal, antibody-independent evidence that LSMEM2 protein is in primary cardiomyocytes and LSMEM2-transfected 293T cells but is not detected in these three non-cardiomyocyte cell types (Extended Data Fig. 7). These data highlight the importance of experimental evidence of surface localization and *N-*glycosylation for defining cell surface *N*-glycoproteins on specific cell types and prioritizing proteins of interest. If we had relied only on database annotations and computational predictions, LSMEM2 would not have been an obvious candidate for further investigation. CellSurfer results, therefore, are invaluable for confirming surface localization, *N-*glycosite and transmembrane topology for uncharacterized or mischaracterized proteins.

### Region-restricted cardiomyocyte cell surface proteins

**Fig. 5:**
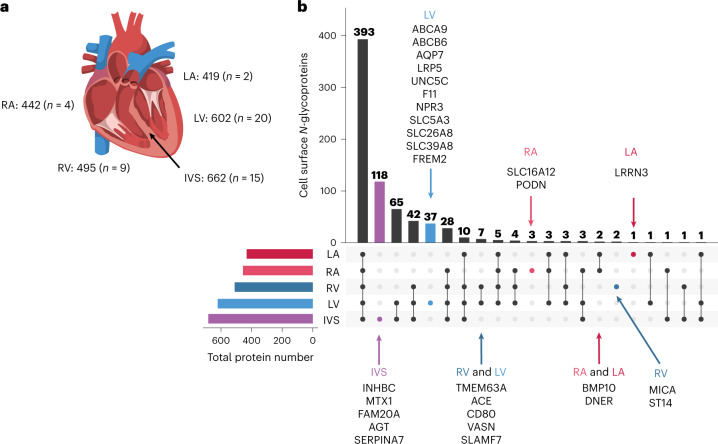
Region-restricted cardiomyocyte cell surface *N-*glycoproteome. **a**, Schematic of the human heart and the number of cell surface *N-*glycoproteins identified for human primary cardiomyocytes isolated from LV and RV, LA and RA and IVS. The number of independent μCSC experiments (*n*) for each cell type is indicated. **b**, UpSet plot visualizing the intersections of identified proteins from human primary cardiomyocytes isolated from LV, RV, LA, RA and IVS. Proteins observed uniquely in one or two regions are listed by gene name. Parts of this figure were generated with BioRender.

There are functional distinctions among cardiomyocytes located in different regions of the heart. Disease processes such as atrial fibrillation and left-sided versus right-sided heart failure affect cardiac cells within specific regions, although current pharmacotherapies for these may not specifically target the affected region-specific cell types, resulting in adverse outcomes (for example, the SCN5A blocker flecainide is used to treat atrial fibrillation but can induce ventricular arrhythmias^22^). Consequently, detailed views of the surfaceome of cardiomyocytes originating from different regions of the heart are needed to inform the development of region-specific drug delivery targets to minimize adverse side effects exerted on cardiomyocytes residing in healthy or unaffected regions. Of 730 cell surface *N-*glycoproteins in cardiomyocytes, voltage-gated calcium channel subunit CACNA1C was detected in five regions (left atrium (LA), right atrium (RA), left ventricle (LV), right ventricle (RV) and intraventricular septum (IVS); Fig. 5a and Supplementary Table 8), consistent with expectations^23^. Voltage-gated sodium channel SCN5A was also detected in cardiomyocytes from all five myocardial regions, which may explain the adverse effects of flecainide on the ventricle. Altogether, 118, 37, 3, 2 and 1 proteins were uniquely identified in IVS, LV, RA, RV and LA cardiomyocytes, respectively (Fig. 5b and Supplementary Table 8). Among these, BMP10 was uniquely detected in LA and RA (Fig. 5b), as expected^24^. Of the 256 proteins observed exclusively in cardiomyocytes in this study, 1, 2, 20 and 64 were detected in cardiomyocytes residing in the LA, RA, LV and IVS, respectively, including LNNR3 (LA), SLC16A2 (RA), SLC26A8 (LV), ABCB6 (LV) and INHBC (IVS) (Supplementary Table 9).

### Stem cell derivatives differ from primary cells

**Fig. 6:**
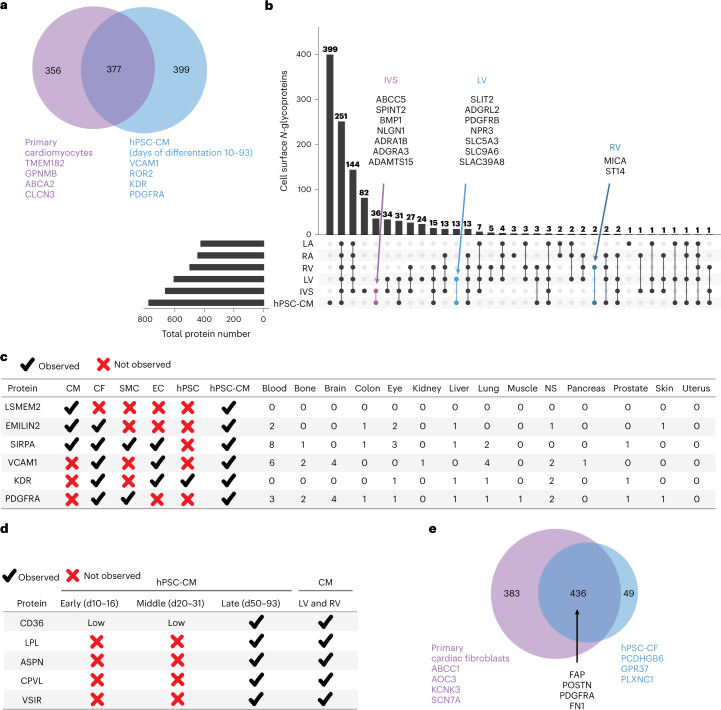
Comparison of hPSC derivatives and primary cardiac cell surface *N-*glycoproteomes. **a**, Venn diagram showing the number of proteins identified in primary cardiomyocytes and hPSC-CMs^17^. Examples of proteins detected only in primary cardiomyocytes or hPSC-CMs are listed by gene name. **b**, UpSet plot visualizing the intersections of identified proteins from human primary cardiomyocytes isolated from LV and RV, LA and RA and IVS and hPSC-CMs. Proteins uniquely shared between different cardiomyocyte regions and hPSC-CMs are listed by gene name. UpSet plot was generated using UpSetR^76^. **c**, Table highlighting detection of previously proposed markers of primary cardiomyocytes (CMs), cardiac fibroblasts (CFs), smooth muscle cells (SMCs), endothelial cells (ECs), hPSCs and hPSC-CMs. The number of cell types from non-cardiac organs for which the protein was observed is indicated. NS, nervous system. **d**, Table highlighting proteins detected in late-stage hPSC-CMs and their presence in primary CMs isolated from LV and RV. **d**, Venn diagram showing the number of proteins identified in primary CFs and hPSC-CFs. Previously published markers of CFs identified in both sample types are listed by gene name.

Human pluripotent stem cell derivatives are an alternative source to primary human heart cells for studying cardiac disease and drug toxicity; however, cellular heterogeneity and relative immaturity compared to primary cells pose challenges^25,26^. Previously, we identified 774 cell surface *N-*glycoproteins by classic CSC across hPSC-CMs (differentiation days (DDs) 10–93^17,18^). Of the 377 proteins detected in both primary cardiomyocytes and hPSC-CMs, 36, 13 and 2 were uniquely shared between hPSC-CMs and IVS, LV and RV cardiomyocytes, respectively (Fig. 6b). As examples, ADGRL2 was observed only in hPSC-CMs and LV, whereas ST14 was detected only in hPSC-CMs and RV cardiomyocytes. We did not identify any proteins that were uniquely present in hPSC-CMs and LA and RA cardiomyocytes, consistent with the differentiation of predominantly ventricular-like hPSC-CMs^27,28^. Of 774 cell surface *N-*glycoproteins identified in hPSC-CMs, 399 were not identified in primary cardiomyocytes from any region (Fig. 6a,b). These include the hPSC-CM marker VCAM1 (ref. 29) and cardiac progenitor cell markers ROR2, KDR and PDGFRA^30^ (Fig. 6c). Conversely, of 730 proteins identified in primary cardiomyocytes across all regions, 356 were not detected in hPSC-CMs (Fig. 6a,b), including 81 drug targets (DrugBank). An example of a drug target for cardiac disease detected in primary cardiomyocytes but not in hPSC-CMs is KCNE1 (azimilide), which has implications for FDA-mandated drug testing for blocking of the HERG channel, because of the risk to patients for torsades de pointes, a life-threatening polymorphic ventricular tachycardia. Moreover, drug targets SCN1B and BACE1, which regulate voltage-gated sodium channels and are critical for normal cardiac conduction^31,32^, were observed in primary cardiomyocytes but not in hPSC-CMs.

The hPSC-CM surfaceome is dynamic with time in culture^17,18^, which critically impacts the utility of hPSC-CMs for drug testing. Four proteins (LPL, ASPN, CPVL and VSIR) identified in primary LV and RV cardiomyocytes were not detected in early-stage (days 10–16) or middle-stage (days 20–31) hPSC-CMs but were present in late-stage (days 50–93) hPSC-CMs (Fig. 6d). Of these, lipoprotein lipase (LPL) is a drug target for cardiovascular disease^33^. These observations exemplify why in vitro derived cells of the appropriate maturation stage will be critical for drug testing and disease modeling. Only cells that express the target for the drug being tested are useful; otherwise, investigators may falsely conclude that a pharmacological agent does not have cardiotoxic effects merely because the full repertoire of receptors present in adult cardiomyocytes is not recapitulated in the tested hPSC-CMs.

Human pluripotent stem cell models of cardiac fibroblasts (hPSC-CFs) also are gaining appeal for drug discovery and disease modeling^34^. Similarly to hPSC-CMs, hPSC-CFs exhibit an embryonic phenotype^34^. Here, comparison of hPSC-CFs to primary cardiac fibroblasts revealed that 436 cell surface proteins were shared among them, including known cardiac fibroblast markers (for example, FAP, POSTN, FN1 and PDGFRA; Fig. 6e). In total, 383 cell surface proteins were unique to primary cardiac fibroblasts, including 85 drug targets (DrugBank). Examples of drug targets for cardiac diseases detected in primary cardiac fibroblasts but not in hPSC-CFs include AOC3 (hydralazine) and NPR2 (nesiritide).

**Fig. 7:**
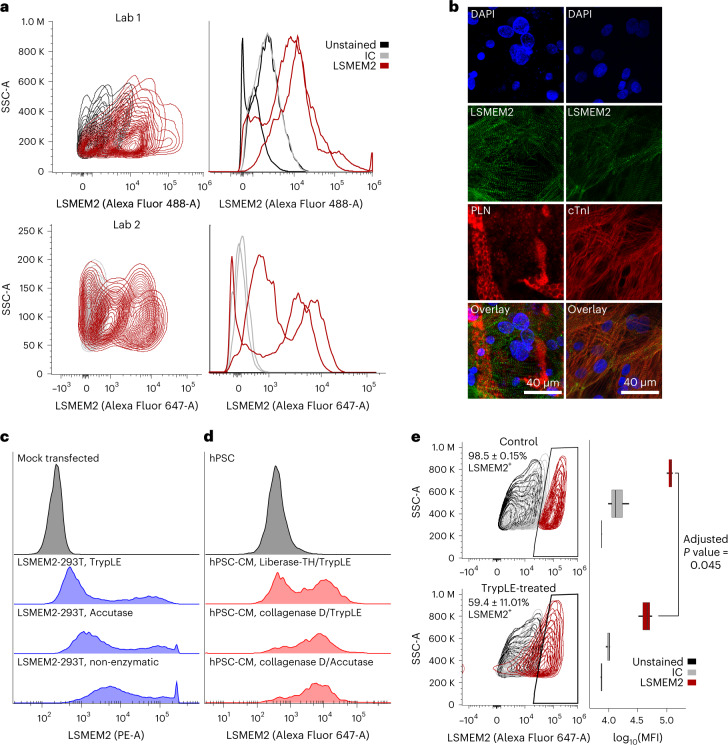
Ruby10.1 anti-LSMEM2 mAb can be used to analyze hPSC-CMs and primary cardiomyocytes. **a**, Live cell flow cytometry analysis of day 30 hPSC-CMs from laboratory 1 (top) and day 14–21 hPSC-CMs from laboratory 2 (bottom). Red traces reflect two biological replicate experiments per laboratory. **b**, Representative immunofluorescence images of day 30 hPSC-CMs co-cultured with adipocytes and co-labeled with Ruby10.1 and anti-PLN or anti-cTnI (representative of three biological replicates). **c**,**d**, Live cell flow cytometry analysis of 293T cells overexpressing LSMEM2 (**c**) and hPSCs compared to hPSC-CMs (**d**) dissociated with various enzyme treatments before antibody labeling. One replicate for each treatment. **e**, Live cell flow cytometry analysis of cardiomyocytes isolated from non-failing human IVS with and without treatment with TrypLE for 5 minutes before antibody labeling. Box plot shows mean fluorescence intensity (MFI) for three technical replicate analyses of control (top) and TrypLE-treated (bottom) (representative of two biological replicates). Box plot shows median with upper lower quartiles; whiskers are 1.5× interquartile range; and *P* values were determined by two-sided *t*-test subsequently adjusted with the Benjamini–Hochberg method (adjusted *P* values for isotype control and unstained are 0.41 and 0.56, respectively). IC, isotype control. Source data

Cell surfaceome data represent a rich source of biomarkers to identify or select cells of a specific cell type or region. Comparisons with primary cell types reveal that hPSC-CM markers SIRPA, EMILIN2 and VCAM1 (refs. 2, 35, 36), which are used alone or in combination (for example, SIRPA/VCAM1 (refs. 35, 37)), are not unique to cardiomyocytes (Fig. 6c). We do not observe VCAM1 on primary cardiomyocytes. In contrast, we observe LSMEM2 uniquely on primary human adult cardiomyocytes and hPSC-CMs^17,18^. To assess the utility of our anti-LSMEM2 mAb for research studies, three laboratories evaluated Ruby10.1 mAb for assessing hPSC-CMs derived from different cell lines and differentiation methods. Two laboratories assessed day 14–30 hPSC-CMs by flow cytometry and found Ruby10.1 suitable for live cell flow cytometry (Fig. 7a). A third laboratory assessed day 30 hPSC-CMs co-cultured with adipocytes using mAb to LSMEM2 and perilipin, a marker for adipocytes, and found these antibodies to exclusively detect hPSC-CMs and adipocytes, respectively (Fig. 7b). Finally, as studies that use hPSC-CMs and cardiac tissue for research commonly use proteases to dissociate cells and tissues, we explored the effect these enzymes have on the ability to detect LSMEM2 on the cell surface. Using 293T cells overexpressing LSMEM2 and hPSC-CMs, we observe that treatment with Accutase, TrypLE or Liberase-TH reduces the detectable signal for LSMEM2 (Fig. 7c,d). Also, treatment of isolated primary human cardiomyocytes for just 5 minutes with TrypLE reduces fluorescence intensity for Ruby10.1 by approximately 60% (Fig. 7e). These results underscore why primary cell isolation and hPSC-CM dissociation methods using collagenases with minimal tryptic activity are required to preserve some extracellular epitopes when dissociating tissues and cells.

### Cardiomyocyte surfaceome changes in heart failure

**Fig. 8:**
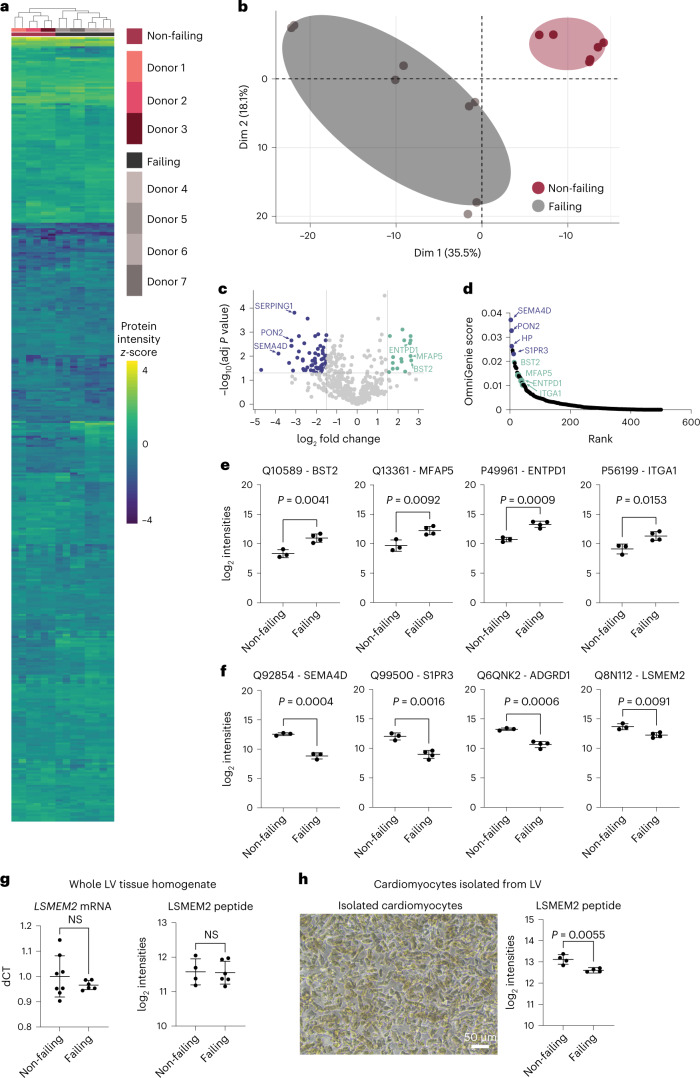
CellSurfer reveals differences in the surfaceome of non-failing and failing cardiomyocytes. **a**,**b**, Heat map (Euclidean distance with complete linkage) representation (**a**) and principal component analysis of quantified cell surface *N*-glycoproteins of non-failing and failing cardiomyocytes (**b**). **c**, Volcano plot highlighting proteins that are differentially abundant in non-failing and failing cardiomyocytes. Purple dots show proteins that were decreased in failing cardiomyocytes, whereas green dots show proteins that were increased in failing cardiomyocytes. Examples of proteins are listed as gene names. Horizontal bar represents a significance level of adjusted *P* ≤ 0.05. Vertical bars represent a log_2_ fold change ≥1.5 (right) and ≤−1.5 (left). The Benjamini–Hochberg procedure was used to calculate adjusted *P* values. **d**, Scatter plot ranking proteins based on OmniGenie score^10^. Purple dots highlight proteins that were decreased in failing cardiomyocytes, whereas green dots highlight proteins that were increased in failing cardiomyocytes. **e**,**f**, log_2_ intensity values are shown for proteins that were higher (**e**) or lower (**f**) in abundance in failing compared to non-failing cardiomyocytes. **g**, qRT–PCR of *LSMEM2* mRNA and targeted MS of LSMEM2 peptide using whole tissue homogenate of LV. **h**, Representative bright-field image of isolated cardiomyocytes from LV and corresponding targeted MS of LSMEM2 peptide from isolated cardiomyocytes from failing and non-failing donors. Proteins are reported by UniProt accession and gene name. Three and four distinct biological replicates for non-failing and failing samples, respectively, for **e** and **f**; eight and six distinct biological replicates for non-failing and failing, respectively, for qRT–PCR and four and six distinct biological replicates for non-failing and failing, respectively, for MS in **g**; and four biological replicates for each condition in **h**. For **e**–**h**, all scatter plots are shown with mean and s.d.; unpaired two-tailed *t*-test unadjusted *P* values are shown. NS, not significant. Source data

Drug development, especially for patients with chronic heart failure^38^, has had limited success over the past decade. In the present study, we applied CellSurfer to cardiomyocytes isolated from non-failing and failing human hearts to detect surface protein differences, to identify potential targets for drug development and to determine whether these differences would contribute to our understanding of the pathophysiological mechanisms. In total, 490 cell surface *N-*glycoproteins were quantified in this comparison (Supplementary Table 10). Hierarchical clustering and principal component analysis group samples by condition and demonstrate that CellSurfer is sensitive enough to distinguish protein differences among patient groups (Fig. 8a,b). The volcano plot highlights cell surface proteins that are differentially abundant between failing and non-failing cardiomyocytes, and analysis by SurfaceGenie^10^ reveals prioritized candidates based on signal dispersion and abundance levels (Fig. 8c,d). Twenty percent (101/490) of quantified cell surface *N-*glycoproteins are significantly differentially abundant between these two patient groups (5% false discovery rate). Previous transcriptomic analyses reveal that 5% of all transcripts are differentially regulated in heart failure^39^. The high proportion of cardiomyocyte surfaceome changes that we observe provides detailed protein-level evidence consistent with previous observations that genes regulating cell–cell and cell–extracellular matrix (ECM) adhesion are generally perturbed in cardiomyopathy^39^. Of the proteins that change in abundance in cardiomyocytes from failing heart (Fig. 8e,f), SEMA4D (ref. 40) and MFAP5 (ref. 41) have been associated with cardiovascular disease but not yet attributed specifically to cardiomyocytes or heart failure. ENTPD1 has been linked to protection from ischemia injury in mouse models, although activity specific to cardiomyocytes has not been elucidated^42^. ADGRD1 and BST2 have not been previously associated with cardiomyopathies and/or heart failure and represent targets for elucidating pathophysiological mechanisms and future drug development.

Among the proteins with decreased abundance in failing cardiomyocytes are S1PR3, which has been shown to protect the myocardium from ischemia–reperfusion injury and represents a therapeutic target in heart failure^43^, and the cardiomyocyte-restricted protein LSMEM2 (Fig. 7f). To determine if this observation for LSMEM2 is potentially due to changes in glycosylation that could affect detection of this protein by µCSC, we performed qRT–PCR using whole tissue lysate and targeted MS analyses of a non-glycosylated LSMEM2 peptide using whole tissue lysate and isolated cardiomyocytes. Significant changes were not observed when analyzing whole tissue homogenate by qRT–PCR or targeted MS of the peptide (Fig. 8g). In contrast, targeted MS analysis shows a significant decrease in LSMEM2 in isolated cardiomyocytes from failing hearts compared to non-failing hearts (Fig. 8h). Notably, the donor samples used for targeted MS differed from those used for µCSC, which adds confidence to the conclusion that LSMEM2 protein is decreased in failing cardiomyocytes. These results highlight challenges when relying on measurements from whole tissue homogenate for organs that are highly heterogeneous. Under conditions where cardiac tissue composition changes (for example, cardiomyocyte death, increased ECM and infiltration of immune cells), normalizing results to total protein will obfuscate results, because it is not possible to control for changes in the cell types or ECM that contribute to the overall total protein content. This could explain why changes for LSMEM2 and other proteins highlighted in Fig. 8e,f have not been reported in three previous generic proteomic analyses of heart failure that used homogenized human tissue^44–46^. Thus, measuring low-abundance cell surface proteins in isolated cells, as done here with CellSurfer, is critical for developing an accurate understanding of the protein events that occur in heart disease.

## Discussion

We generated experimentally derived surfaceome maps of primary human heart cells. The human cardiac cell surfaceome (1) provides critical information for updating protein database annotations and prioritizing drug targets, including cell-type-restricted and region-restricted cell surface glycoproteins; (2) provides unprecedented insight and empirical evidence of differences between cardiac cells at a molecular level inaccessible to transcriptomics and typical proteomic approaches; (3) contains experimentally validated extracellular epitopes that inform topology for transmembrane proteins and facilitate the development of an mAb targeting a previously unknown, but highly selective, cardiomyocyte protein; (4) reveals key dissimilarities between pluripotent stem cell derivatives and their primary cell counterparts, including drug targets; and (5) exposes previously uncharacterized differences in the surfaceome of cardiomyocytes in failing and non-failing hearts.

The surfaceome maps were achieved through the development of CellSurfer, an analytical platform for reproducible, quantitative discovery of cell surface *N-*glycoproteins from <0.3–10 million cells or 100–1,000 μg total peptides in under 45 hours, which is an ~90% and ~50% reduction in starting material and time, respectively, compared to previous approaches^47,48^. Our webtool, Veneer, provides an automated, standardized solution for classification, annotation and accurate reporting of data generated by μCSC and related workflows. Whereas most cell surface proteins are potentially glycosylated^9,49^, μCSC is biased to detecting *N-*glycosylated peptides that contain available and accessible vicinal-diol groups on corresponding glycans and for which the glycosite resides in a tryptic peptide of suitable *m/z* for MS detection^14,47^. Thus, relevant non-glycosylated proteins (for example, TMEM65 (ref. 50) or RARG (ref. 51)) will be undetected. Presently, μCSC does not have the sensitivity to analyze single cells. The analyses thus represent an average view of the cell surface *N*-glycoproteome from a population of cells. As differences in peptide abundance measured by μCSC could be affected by changes in glycan structure, orthogonal data, such as targeted MS of non-glycosylated peptides as we did here for LSMEM2, may be important for distinguishing apparent differences in abundance at the peptide level versus those caused by changes in glycosylation that affect detection by μCSC. In the future, sample preparation methods that generate experimental evidence of surface localization in ways that complement the coverage of μCSC and include information regarding the glycan moiety will further expand our view of the surfaceome. The miniaturized sample handling workflow developed for μCSC and data analysis features of Veneer can easily be adapted for such methods. As CellSurfer is applied broadly to other human cells, public repositories can be increasingly populated with cell-type-specific data to facilitate the identification of cell-restricted and organ-restricted proteins.

Although the protocol to isolate cardiomyocytes and cardiac fibroblasts from cryopreserved tissue represents a technological advance, the limited availability of fresh tissue samples represents a potential study limitation. The total number of µCSC experiments performed depended on the number of cardiac regions available for processing. For qualitative analyses, we opted to make the most of each sample and used the greatest number of samples available and largest total peptide amount possible instead of normalizing all samples by peptide amount, as we did for the quantitative analyses. As the number of samples varied by cell type and by cardiac region, and because isolation procedures may yield some contaminating cell types or cells with compromised plasma membranes, further validation of these proteins is required. Also, as the presence of PDGFRA and POSTN in cardiac fibroblasts indicates an activated phenotype^52^, future studies that profile non-activated fibroblasts are warranted. Despite these constraints, many cell-type-restricted cell surface proteins identified here are consistent with expectations (for example, SCN5A^23^, CACNA1C^23^ and PDGFRA^52^).

**Extended Data Fig. 8:**
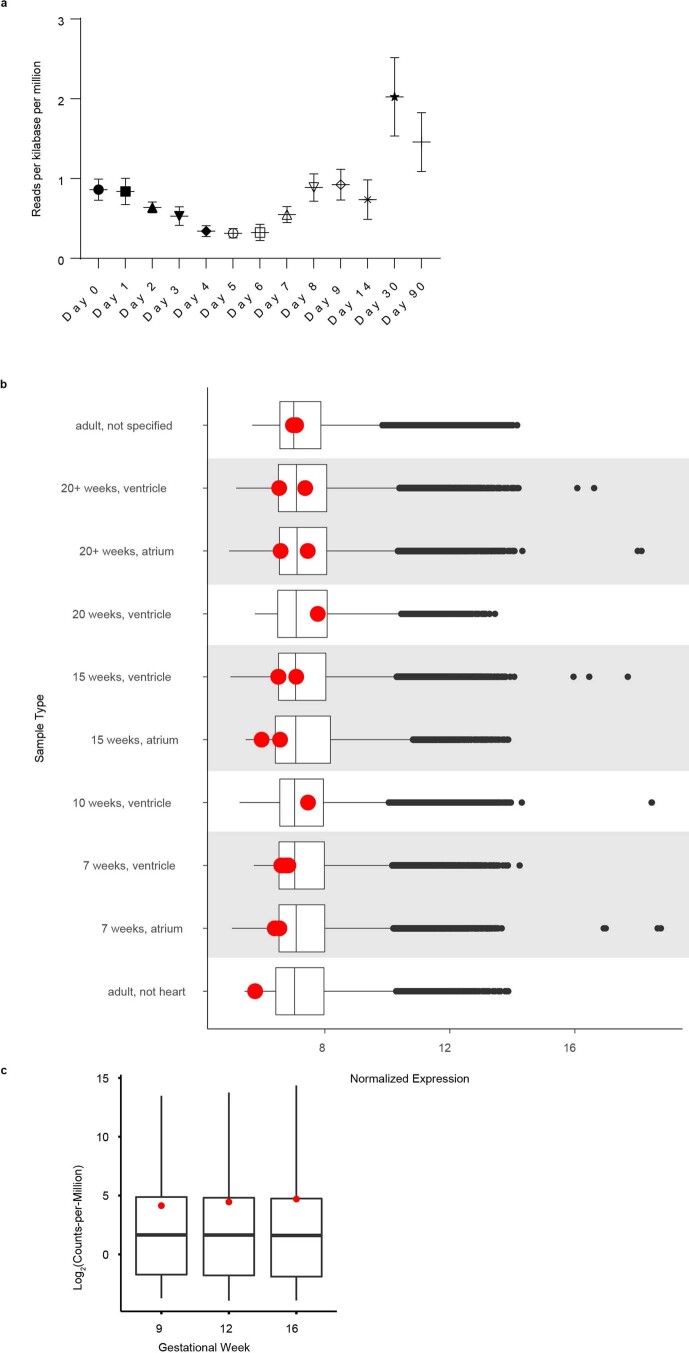
LSMEM2 expression during hPSC-CM differentiation and human heart development. All data are from previously published studies **a**. RNA-seq analysis of LSMEM2 expression during hPSC-CM differentiation^28^. 3 separate differentiations and RNA-seq performed on each day, data show mean with SEM. **b**. Microarray^53^ and **c**. RNA-seq analysis of LSMEM2 expression during human heart development^54^. Boxplots in **b** and **c** display median and interquartile range (upper and lower) and whiskers are 1.5X interquartile range and outliers are shown. For **b**, raw counts were accessed from ebi.ac.uk (E-MTAB-7031). Counts per million (CPM) were calculated for all genes, the mean was calculated for each gestational stage (n = 3 for each) and plotted as a boxplot. LSMEM2 CPM was overlaid onto this boxplot shown as a red point. For **c**, normalized data were accessed from ebi.ac.uk (E-GEOD-71148). The mean was calculated for each sample type and plotted as a boxplot. LSMEM2 expression was overlaid onto this boxplot shown as a red point. N for sample types (top to bottom) are 3, 2, 2, 1, 3, 2, 1, 3, 2, 1.

We provide orthogonally validated evidence that LSMEM2, among others, is a cell surface *N-*glycoprotein on human cardiomyocytes. LSMEM2 transcripts are detected early and do not significantly change during hPSC-CM differentiation or during human heart development (Extended Data Fig. 8)^28,53,54^. The detection of LSMEM2 at intercalated discs suggests a functional role either in cell–cell junctions or in the chemo-electro-mechanical coupling of cardiomyocytes. Based on phylogenetic analysis, LSMEM2 is predicted to be present only in high-order vertebrates with complex hearts (three chambers with septation or four chambers). In fact, the presence of low-complexity regions, such as those predicted within LSMEM2, is correlated with the emergence of novel protein function in higher-order eukaryotes^55^. The high degree of disorder in LSMEM2 structure predictions, in tandem with the low-complexity regions, late evolutionary emergence and restricted expression profile, suggest that it might be a protein involved in multivalent interactions important for the organization of molecular complexes within heart. Finally, LSMEM2 does not contain a canonical signal peptide and was not predicted to be surface-localized in three previous human prediction studies^1,56,57^. Thus, if we had relied only on computational predictions or public repository annotations, LSMEM2 would never have been prioritized as a potential cardiomyocyte surface marker.

The use of biomarker combinations that enable precise identification and functional characterization of cell-type-specific, maturation-stage-specific and region-specific hPSC-CMs or for sorting primary cell types will promote their utility for accurate drug testing, modeling and treatment of chamber-specific diseases. In this context, we expect LSMEM2 to be useful as a quality control metric to evaluate the phenotypic authenticity of hPSC-CMs, as it was detected on primary cardiomyocytes from LV, RV, IVS, LA and RA. Specifically, if hPSC-CMs lack LSMEM2 (that is, a marker of authentic cardiomyocytes found in the human heart), this would indicate that the model does express the full repertoire of surface proteins found in the primary cardiomyocyte phenotype. We predict that markers that are cardiomyocyte specific (for example, LSMEM2) when combined with maturation stage (for example, CD36 (ref. 17)) and region-specific markers will be powerful tools for selecting and characterizing functionally defined hPSC-CMs to promote their use in research and clinical applications. Finally, we observed that LSMEM2 is sensitive to proteases commonly used to dissociate cells and tissues in research applications (for example, passaging hPSC-CMs and dissociating tissue to isolate cardiomyocytes); therefore, the presence of LSMEM2 will be a useful quality control metric to assess whether the cell preparation or isolation method preserves the full repertoire of surface proteins.

Crucially, considerable differences were observed between the surfaceome of hPSC derivatives and their primary cell counterparts. In cases where hPSC derivatives that lack cell surface proteins present in primary cells are used in drug testing, false-negative results would be expected. Differences in functional attributes between hPSC-CMs and adult cardiomyocytes such as channels or enzymes (for example, KCNE1, SCN1B and BACE1) involving tests for anti-arrhythmic and pro-arrhythmic drugs would also be contraindicated. On a positive note, comparative surfaceome approaches have led to the discovery of targets for drug and gene therapy in undifferentiated pluripotent stem cells and primary human fetal retinal pigmented epithelial cells^58,59^. Ultimately, knowledge of surfaceome discrepancies between hPSC derivatives and primary cells can be exploited to monitor or drive hPSC derivatives toward more subtype-specific adult phenotypes. This approach has been applied to hepatocytes^60^, and we expect the same for cardiac cells.

Finally, as efforts to develop therapeutic targets for heart failure shift away from secondary effects (for example, neurohormonal activation and renal function) and toward combating the development and progression of structural cardiac abnormalities associated with heart failure^38^ (for example, hypertrophy, fibrosis and abnormal calcium handling), analysis of the surfaceome will be critical to advances in the cardiovascular field given its critical role in structural integrity, cell communication (direct, electrical and hormonal), immunity and cell protection. In this study, we demonstrated that application of the CellSurfer platform to isolated cells provides a level of molecular detail not revealed by generic proteomic or transcriptomic analyses of whole tissue analyses and, thus, will be critical for carefully mapping the molecular changes in disease. For example, we observed that the abundance of LSMEM2 in cardiomyocytes decreases with heart failure. Although the future utility of LSMEM2 as a payload delivery target is promising due to its restricted expression to cardiomyocytes, its potential as a direct target of a drug to modulate cardiomyocyte physiology will depend on its function, which is yet unknown. Finally, we identified known drug targets for non-cardiac diseases, including those that have been associated with adverse cardiac outcomes (for example, ABCC1 (target of abiraterone for prostate cancer) and DPP4 (target of sitagliptin for pancreatic beta cells in diabetes)). As new drug targets for non-cardiac disease are identified, this resource can be rapidly screened to prioritize targets that are predicted to avoid cardiotoxicity. In conclusion, the cardiac surfaceome differs among cell types, and it changes in development and disease. By defining these differences, surfaceome-based biomarkers will advance the development of cell-type-specific and organ-specific targets for drug delivery, disease modeling and immunophenotyping, and it represents the first steps toward understanding how surface proteins contribute to region-restricted (patho)physiological mechanisms in the human heart.

## Methods

### Cell culture

Undifferentiated hPSC (DF6-9-9T, JHU001) and hESC (H7 line) were maintained in monolayer culture. Differentiation into hPSC-CMs was performed as described^17,27,28^. The hESC line H7 was cultured in E8 media until 80% confluent. CHIR99021 (4 µM; Selleck Chemicals) was added for 2 days, followed by 1 day of rest. IWR-1-endo (5 µM; Selleck Chemicals) was added for 2 days and maintained in RPMI-B27 until used for analysis (days 25–30). The hiPSC line DF6-9-9T was maintained on Matrigel-coated dishes in E8 medium until fully confluent. Cells were switched to RPMI 1640 (with L-glutamine) media with 2% B27 minus insulin supplement. Differentiation was initiated by the addition of CHIR99021 (6 µM final; Selleck Chemicals) on days 0–1 and followed by treatment of IWR-1 (5 µM final; Selleck Chemicals) on days 4–5. From day 8 of differentiation on, cells were fed with RPMI 1640 (with L-glutamine) with 2% B27 plus insulin supplement and 2% FBS. The hiPSC line JHU001 was maintained on Geltrex-coated (Life Technologies) dishes in E8 medium containing supplements (Thermo Fisher Scientific). On DD0, the medium was changed from E8 to RPMI medium supplemented with B27 lacking insulin (Thermo Fisher Scientific). CHIR99021 (6 μM) was added to the cells from DD0 to DD2, followed by the addition of IWR-1 (10 μM; Selleck Chemicals) from DD3 to DD5. After DD7, cultures were maintained in RPMI/B27 medium containing insulin (Thermo Fisher Scientific). Cells were passaged on DD10–DD14 using TrypLE and replated in RPMI/B27 supplemented with the ROCK inhibitor Y27632 (Tocris; 1 mM). Replated hiPSC-CM cultures were incubated for 4 days in glucose-depleted DMEM medium supplemented with 4 mM L-lactate (lactate medium; MilliporeSigma). Cells were re-passaged with TrypLE at DD20–D25 and 7 days before experimentation. Frozen aliquots of human adipose stem cells (hASCs) were thawed, expanded and differentiated to the adipogenic lineage as described^61^. In brief, hASCs were cultured in DMEM medium (Life Technologies) supplemented with 10% FBS (MilliporeSigma), 0.5% penicillin–streptomycin (Cellgro), 1 ng ml^−1^ of FGF2 (PeproTech) and 0.5% antibiotic/antimycotic agent. hASCs at passages 3–4 were plated at a density of 20,000 cells per cm^2^. After 48 hours, cells were cultured in medium consisting of high-glucose DMEM, 10% FBS (MilliporeSigma), 200 mM indomethacin (MilliporeSigma), 500 mM 3-isobutyl-1-methyl xanthine (IBMX) (MilliporeSigma), 1 mM dexamethasone (MilliporeSigma), 5 mg ml^−1^ of human insulin (MilliporeSigma) and normocin (Invivogen) for 14 days, with medium changes every 2 days. After differentiation, the cells could be passaged and plated with CMs in RPMI/B27 + insulin medium for at least 7–10 days. Differentiation of hiPSCs into hPSC-CFs was performed as described^34^ using three hiPSC lines (UAZTi004-A, UAZTi005-A and UAZTi006-A) obtained from the University of Arizona iPSC Core. In brief, hiPSC lines were cultured in E8 until reaching 80% confluency. iPSCs were treated with 4 µM CHIR99021 (Selleck Chemicals) for 2 days, followed by a day of rest in base media (RPMI with B27 minus insulin). Cells were cultured in CFBM media (75 ng ml^−1^ of FGF2 (PeproTech)) until day 20 and passaged. hiPSC-CFs were cultured for an additional 6 days before freezing in 20% DMSO in CFBM media. Cells were thawed and plated in IMDM and cultured for 6 days before use for µCSC. Vendor and catalog information for all reagents and cell lines are provided in Supplementary Table 11. Primary coronary artery smooth muscle cells and cardiac microvascular endothelial cells were obtained commercially. 293T, RPMI 1788, HeLa, U2-OS and Jurkat cells were cultured with DMEM, IMDM, EMEM, McCoy’s 5A medium and RPI 1640, respectively, modified with 100 U ml^−1^ of penicillin, 100 μg ml^−1^ of streptomycin and 10% FBS. All cells were maintained in a humidifying atmosphere at 5% CO_2_ and 37 °C. All purchased cell lines were authenticated by short tandem repeat profiling and shipped with authentication certificates from the vendor. Cardiac microvascular endothelial cells were cultured in EBMTM-2 with 100 U ml^−1^ of penicillin, 100 μg ml^−1^ of streptomycin, 10% FBS and SingleQuots supplement as recommended by the manufacturer. Coronary artery smooth muscle cells were cultured in vascular cell basal media with 100 U ml^−1^ of penicillin, 100 μg ml^−1^ of streptomycin and 10% FBS. Endothelial cells and smooth muscle cells were received at passage 3 and passaged until passage 6 (endothelial cells and smooth muscle cells) and passage 8 (endothelial cells). Co-cultures of cardiomyocytes and adipocytes were maintained in cardiomyocyte maintenance medium (RPMI supplemented with B27 + insulin).

### Donors used for cardiomyocyte and cardiac fibroblast isolation

Human cardiac tissue used for cardiomyocyte and cardiac fibroblast isolation was obtained as de-identified material from institutional tissue banks under institutional review board approvals (PRO00025506 and PRO643-17-EP) at the Medical College of Wisconsin and the University of Nebraska Medical Center. Informed consent was obtained from all donors, and no compensation was provided to study participants. The number of samples, de-identified donor information (sex, age, ethnicity and medical history) and heart regions used for all µCSC experiments of non-failing hearts are provided in Supplementary Table 12. Samples used for µCSC, qRT–PCR and targeted MS analyses of failing and non-failing hearts are in Supplementary Table 13.

### Isolation of primary cardiomyocytes from cryopreserved human heart tissue for µCSC

Cardiac tissue was collected 5–48 hours after harvest based on time to complete surgery. Occasionally, the heart was transferred to the pathology department for evaluation before we could access it (48 hours). Upon receipt in the research laboratory, all cardiac tissue was immediately cryopreserved in 10% DMSO, 10% FBS and 80% DMEM until use. Primary cardiomyocytes were isolated using a recently developed method that enables isolation of cardiomyocytes in a manner that preserves plasma membrane proteins^62^. In brief, tissue pieces were thawed on ice, collected with a 70-μm cell strainer and washed three times with ice-cold wash solution (115 mM potassium gluconate, 2.5 mM potassium chloride, 5 mM potassium phosphate monobasic, 2 mM magnesium sulfate, 2 mM calcium chloride, 30 mM sucrose and 10 mM 4-(2-hydroxyethyl)-1-piperazineethanesulfonic acid, pH 7.4). Tissues were sliced using a Leica VT1200S vibrating blade microtome while submerged in oxygenated ice-cold wash solution. Tissue slices were placed in digestion solution (300 U ml^−1^ of collagenase type 4, 5 U ml^−1^ of DNase I and 1 mg ml^−1^ of trypsin inhibitor in wash solution) in a 50-ml spinner flask at 37 °C. Crude cardiomyocytes were filtered through a 200-µm cell strainer and collected by centrifugation at 80 *g* for 5 minutes at 4 °C and washed three times in ice-cold wash solution. Cellular debris was separated from the crude cardiomyocytes by Percoll gradient as described^62^. Cardiomyocytes were isolated from other cell types using human CD31 and human CD45 Dynabeads by the process of negative selection^62^.

Cardiomyocytes were intact after isolation and exhibited rod-shaped morphology (Extended Data Fig. 4), and cell surface biotinylation confirmed that membrane integrity was preserved before cell lysis (Extended Data Fig. 4c). This cardiomyocyte isolation method yields ≥80% troponin I3-positive cardiomyocytes^62^ based on flow cytometry comparison of antibody to isotype control. More than 95% purity is estimated if comparing to unstained control. Although we routinely detect common non-myocyte cell markers CD105 (endothelial cells), CD45 and CD11b (immune cells) in whole tissue homogenate^62^, we did not identify these proteins in isolated cardiomyocytes when comparing failing and non-failing hearts (Supplementary Table 10). Although some non-cardiomyocytes may be present in isolated cardiomyocytes, the comparison to non-cardiomyocyte cell types provides evidence of proteins that may be cell type specific versus those that are present in multiple cell types.

### Isolation of primary cardiac fibroblast from cryopreserved heart tissue for µCSC

Cardiac fibroblasts were isolated and cultured as described^63–65^. Cryopreserved tissues were thawed and cut into 1-mm^3^ pieces while submerged in HBSS with magnesium and calcium (HBSS^+/+^). Tissue pieces were placed in digestion solution (0.1 U ml^−1^ of collagenase B, 150 U ml^−1^ of collagenase IV, 0.5 mg ml^−1^ of Dispase II and 5 U ml^−1^ of DNase I in HBSS^+/+^) in a 50-ml conical tube at 37 °C while rotating end-over-end. Tissue pieces were collected with a 70-µm cell strainer. Flow-through was collected and centrifuged at 300 *g* for 5 minutes. Cells were resuspended in IMDM with 100 U ml^−1^ of penicillin, 100 µg ml^−1^ of streptomycin, 0.25 µg ml^−1^ of amphotericin B and 20% FBS and plated on 0.2% gelatin. Morphology of cardiac fibroblasts was assessed by immunofluorescence microscopy (Extended Data Fig. 4e).

### Transfection of 293T cells for overexpression of LSMEM2

293T cells were transfected with polyethylenimine using a modified version of a published protocol^66^. In brief, cells were plated at a density of 8 × 10^4^ cells per cm^2^ in a 10-cm plate on the day of transfection. Then, 5.25 µg of DNA was pre-incubated in 1 ml of 0.24 mM polyethylenimine in 150 mM NaCl for 10 minutes and then added to 293T cells. Mock-transfected cells followed the same protocol except for the absence of DNA. GFP transfection was performed in parallel to each LSMEM2 transfection to validate successful handling of the cells and preparation of transfection complexes. Cells were fed with fresh media on day 1 after transfection and were collected on day 2. LSMEM2 vector was purchased from Dhamacon. GFP vector was either a gift from Blake Hill (Medical College of Wisconsin) or purchased from Addgene.

### μCSC workflow

Major features of μCSC that distinguish it from classic CSC^47^ include: (1) a catalyst is included in the biotinylation reaction to increase labeling efficiency; (2) total cell lysis is performed using ultrasonic vibration in place of plasma membrane rupture, followed by differential centrifugation-based membrane enrichment to minimize sample loss; (3) glycopeptide enrichment is performed using magnetic streptavidin beads to facilitate automation in place of polyacrylamide resin beads in a filter-based format; (4) binding capacity of streptavidin beads is assessed by the AVIDITY assay^67^ to ensure reproducible and high performance; (5) glycopeptide enrichment and post-enrichment bead washing includes harsh detergents (SDS and Tween) to improve removal of non-specific binders; (6) peptide cleanup is achieved using the magnetic-particle-based SP2 protocol^68^ for detergent removal in place of reversed-phase spin columns; and (7) glycopeptide capture and washing are performed using an automated liquid handling workstation to minimize human intervention and maximize reproducibility.

All steps were performed on ice unless otherwise noted, and all solutions were made fresh. Cells were washed twice with room temperature wash solution (1× PBS and 0.1% FBS) and once with cold labeling buffer (1× PBS and 0.1% FBS, pH 6.0) before oxidization with 1 mM sodium-meta-periodate in cold labeling buffer for 15 minutes in the dark at 4 °C. Oxidation solution was removed, and quench solution (2 mM sodium sulfite in cold labeling buffer) was added to cells and incubated for 10 minutes in the dark at 4 °C. Cell were washed twice with cold labelling buffer, followed by incubation in 10 mM biocytin hydrazide and 50 mM (1H-imidazol-2-YI)-methanamine dihydrochloride in cold labeling buffer for 60 minutes in the dark at 4 °C. Cells were washed three times with cold wash buffer (1× PBS and 0.1% FBS). Cells were resuspended in lysis buffer (40% 250 mM ammonium bicarbonate/40% 5× invitrosol/20% 100% acetonitrile) and sonicated using a VialTweeter with three intervals of 10-second pulses with 10 seconds of cooling on ice between repetitions. Proteins were reduced with 5 mM tris(2-carboxyethyl)phosphine for 30 minutes at 37 °C with vortexing, followed by alkylation with 10 mM 2-iodoacetamide for 30 minutes at 37 °C with vortexing. Protein quantitation was determined using the Pierce 660-nm Protein Assay. Trypsin/Lys-C mixture was added at 1:40 enzyme-to-substrate ratio, and pH was adjusted to ~8.5 by the addition of 2N NaOH and digested overnight at 37 °C with vortexing using a thermomixer at 1,200 r.p.m.

Peptide quantitation was performed using Pierce Quantitative Fluorometric Peptide Assay. After peptide quantification, 1 mM *N*_*α*_-tosyl-L-lysine chloromethyl ketone dihydrochloride (trypsin inhibitor) was added and incubated at room temperature for 5 minutes. Then, 135–1,289 μg of peptides (250,000–10 million cells) was diluted in binding buffer (80 mM sodium phosphate, 2 M NaCl, 0.2% Tween 20, 25 µM EDTA and 0.1% SDS, pH 7.8) and boiled for 5 minutes at 95 °C in heat block, followed by cooling on ice for 5 minutes. Next, 100 μl of Streptavidin MagBeads (GenScript, L00424) were equilibrated in binding buffer (80 mM sodium phosphate, 2 M NaCl and 0.2% Tween 20, pH 7.8) and added to the peptides and incubated for 1 hour with mixing. Beads were washed sequentially with (1) 2% SDS in 1× PBS; (2) 80 mM sodium phosphate, 2 M NaCl and 0.2% Tween 20, pH 7.8; (3) 100 mM sodium carbonate; and (4) 50 mM ammonium bicarbonate. Peptides were released by incubating the beads in 40 μl of 50 mM ammonium bicarbonate containing 2 μl of PNGase F overnight at 37 °C with vortexing. Supernatant was removed to a new tube; beads were rinsed once with 20 μl of 0.1% SDS in ultrapure water; and the rinse was combined with the supernatant. Peptides were cleaned using SP2 to remove detergents and other contaminants as previously described^68^. In brief, peptides were mixed with 6 µl of pre-washed carboxylate-coated magnetic particles (50 µg µl^−1^, 1:1 mix of hydrophobic and hydrophilic; GE Life Sciences, 65152105050250 and 45152105050250). Peptides were bound to beads by bringing the mixture to 95% MeCN by the addition of 100% MeCN. After allowing 2 minutes for peptides to bind, particles were collected by magnetic rack, and supernatant was removed. Particles were washed twice with 500 µl of 100% MeCN. Peptides were eluted from particles using 38.5 µl of 2% MeCN in water. Glycopeptide capture and bead washing were performed using an epMotion 5073m (Eppendorf).

Samples were analyzed by liquid chromatography with tandem mass spectrometry (LC–MS/MS) using a Dionex UltiMate 3000 RSLCnano system in line with an Orbitrap Exploris 480 MS, Fusion Lumos or Q Exactive. All methodological details for MS acquisition and analysis are described in Supplementary Table 14. Data were processed with ProteomeDiscoverer 2.4 (Thermo Fisher Scientific), implementing Sequest and MSFragger search algorithms followed by Percolator for post-search validation^69–71^. DIA data were analyzed in Spectronaut 15 (Biognosys) using default settings^72^. Pooled DDA runs were searched separately using the Pulsar search engine to create a spectral library, which was then used to perform spectral matching analyses on DIA runs using default settings, with the exception of single hit definition being set to ‘By Modified Sequence’ and having PTM Analysis checked under the PTM Workflow tab. Quantitative data were further processed in MSstats^73^. Peak intensities were log-transformed, normalized by applying equalize median normalization and summarized using Tukey’s median polish run-level summarization. Censored missing values were imputed by Accelerated Failure Time model. Significantly different proteins were determined by fold change ≥1.5 and ≤−1.5 and adjusted *P* < 0.05. The quantitative cardiomyocyte study was performed with one pooled LA and RA sample (six donors each) and three matched biological replicates for LV and RV. Median percent coefficients of variation for raw intensities of *N*-glycopeptides are 39.7% and 34.8% for LV and RV cardiomyocytes, respectively (Extended Data Fig. 5b). Statistical analyses (Pearson correlation coefficient, percent coefficient of variation and unpaired two-tailed *t*-test) were performed in GraphPad Prism (version 9) and R (version 4.1.1). The CSC data analysis and annotation workflows were built in Python (version 3.6.9) and the R Shiny app.

### Stringent criteria for classification of cell surface *N*-glycoproteins

**Extended Data Fig. 9:**
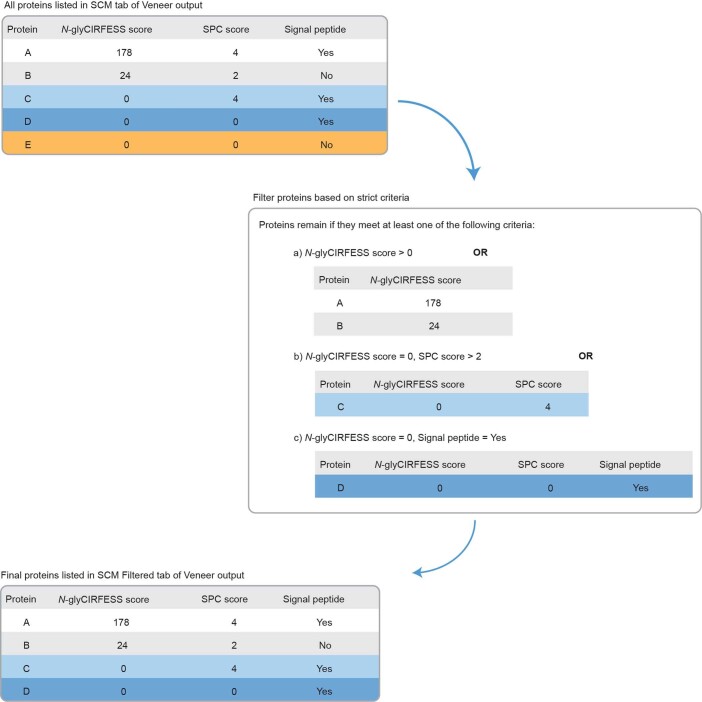
Classification criteria for cell surface *N*-glycoproteins. Proteins remain in the dataset if they have (**a**) a *N*-glyCIRFESS score greater than 0, (**b**) a *N*-glyCIRFESS score equal to 0 and a SPC score greater than 2, or (**c**) a *N*-glyCIRFESS score equal to 0 and a predicted signal peptide motif.

Throughout the study, we considered only unique master protein accessions identified by at least one peptide containing a deamidation within the sequence consensus motif (SCM). Within this subset of proteins, classification as a cell surface *N-*glycoprotein was based on *N*-gly-CIRFESS score, SPC score and signal peptide prediction from three algorithms (PrediSi^74^, SignalP^75^ and Phobius^19^), all of which are based on primary sequence analysis and are reported in the Veneer output (Extended Data Fig. 9). Specifically, identified proteins in the SCM tab of the Veneer output were classified as cell surface *N-*glycoproteins if they were annotated with an *N*-gly-CIRFESS score >0 or if they had an *N*-gly-CIRFESS score of 0 and an SPC score of 3–4 or if they had an *N*-gly-CIRFESS score of 0 and a predicted signal peptide motif. The rationale behind these criteria is that an *N*-gly-CIRFESS score of 0 indicates that there are no predicted extracellular PSMs with SCMs suitable for MS analysis, and, thus, the protein would not be specifically detected by a CSC approach (that is, if it is present in the output, then it is most likely a non-specific binder). However, the false classification rate of transmembrane helices for the Phobius and TMHMM algorithms used in CIRFESS are 7.9%^19^ and 2–3%^20^, respectively. Also, predictions in CIRFESS are limited to extracellular SCM peptides with more than five amino acids and *m/z* of 2+ or 3+ charge state peptide <2,000. Thus, it is possible, although rare, for a cell surface *N-*glycoprotein to have an *N*-gly-CIRFESS score of 0. Therefore, proteins with an *N*-gly-CIRFESS score of 0 and an SPC score of 3 or 4 were considered *N*-glycosylated cell surface proteins because there is high confidence in surface localization of these proteins. Also, for proteins predicted to have a signal peptide motif, there is high likelihood that these proteins are released into the extracellular space. For all analyses in this study, the specificity calculated by Veneer is reported as the number of PSMs or proteins identified with deamidation in the SCM out of the total PSMs or proteins, respectively. The number of cell surface *N-*glycoproteins identified are those proteins from the SCM tab that meet the above-mentioned classification criteria and are reported in the SCM Filtered tab of Veneer output.

### Anti-LSMEM2 monoclonal antibody development and validation

A peptide epitope (CLKLRLASLSQLRRL) from the extracellular domain of LSMEM2 was selected, and antibody development was performed by GenScript using KLH conjugation because of the homology between mouse and human LSMEM2. In brief, three Balb/c mice and three C57 mice were injected with KLH-modified peptide. Ascites was collected and screened by ELISA and live cell flow cytometry. The mice with the highest signal above negative control were used for hybridoma generation. Hybridomas were screened by ELISA first and then, if positive, screened by live cell flow cytometry. Positive hybridomas were subcloned to ensure monogenicity. Isotypes were determined using the Pierce Rapid Antibody Isotyping Kit.

### Immunofluorescent imaging

Fresh-frozen tissue was cut into 20-µm-thick sections using a cryotome, and sectioned slides were kept at −80 °C until use. Slides were allowed to come to room temperature before excess OCT compound was removed by washing the slides in tris-buffered saline solution (5 mM tris-hydrochloride, 1 mM CaCl_2_, 2 mM MgCl_2_ and 125 mM NaCl) with 0.025% Triton X-100 (TBST). A hydrophobic coating was placed around tissues with an ImmEdge Pen. For antigen blocking experiments, primary antibodies were pre-incubated with antigen peptides at 100× the molecular weight excess for 45 minutes at room temperature before use.

Slides and cells were imaged with a Zeiss Laser Scanning Confocal Microscope 880 with Airyscan or a Zeiss 700 or 710 Laser Scanning Microscope. Images were acquired using ZEN Black (Zen 2012 SP5 FP3 14.0.21.201). ZEN Blue (2.3.69.1000) and ZEN Lite imaging software were used to process images. Detectors included LSM800 GaAsP-Pmt2 for AF633 (detection wavelength 636–700 nm), LSM800 Airyscan for AF488 (detection wavelength 495–570 nm) and LSM800 GaAsP-Pm for DAPI (detection wavelength 400–488 nm). We did not use rainbow pseudo-color and did not quantify the information from images. First, deconvolution was performed with default settings in ZEN Blue and then image projection from z-stacks using maximum intensity projection. All images with the scale bar of 50 µm were acquired with ×40 objective EC Plan-Neofluar ×40/1.3 Oil DIC M27, with resolution 1,024 × 1,024 pixels, 16-bit, single plan, no z-stacks. All images with the scale bar of 20 µm were acquired at ×63 objective Plan-Apochromat ×63/1.40 Oil DIC M27, with resolution 1,024 × 1,024 pixels, 16-bit. Series of 16–20 z-stacks with 5–8 µm between each. Images with the scale bar of 5 µm are inserts from the original image. Co-cultures of hPSC-CMs and adipocytes were fixed with 4% paraformaldehyde for 20 minutes at room temperature, followed by three washes with DPBS for 30 minutes each. Samples were permeabilized with 0.2% Triton X-100 for 10 minutes and blocked with 10% normal goat serum (Sigma-Aldrich) for 30 minutes. Samples were incubated overnight with primary antibodies (LSMEM2, cardiac troponin I (United States Biological, T8665-13F) and perilipin (Abcam, ab3526)) diluted in 10% normal goat serum at 4 °C. After three washes with DPBS, samples were incubated with secondary antibodies Alexa Fluor 488-conjugated goat anti-mouse or Alexa Fluor 647-conjugated goat anti-rabbit (Thermo Fisher Scientific). Samples were washed three times with DPBS and incubated with DAPI (Sigma-Aldrich) diluted in DPBS. Fixed and immunostained cells were imaged using a Zeiss LSM 700 Confocal Microscope. For images of hPSC-CMs co-cultured with adipocytes, ×20, ×40 and ×63 objectives on the Zeiss LSM 700 Confocal Microscope were used, with image projection from z-stacks using maximum intensity projection and images processed in Image J.

### Preparation of single-cell suspensions of cell lines for flow cytometry analysis of LSMEM2

To generate single-cell suspensions for 293T, HeLa, U2-OS and Jurkat cell lines and hPSC-CM, cells were washed once with DPBS^−/−^ and dissociated using cell-type-specific conditions. For Ruby10.1 immunodetection, 293T cells were washed twice in DPBS^−/−^ and then incubated in 0.5 mM EDTA in DPBS^−/−^ at 37 °C for 10 minutes. EDTA solution was removed, and cells were further incubated in DPBS^−/−^ for 10 minutes at room temperature before collection by trituration. 293T, HeLa and U2-OS cells were washed twice in DPBS^−/−^ and then incubated in Cell Dissociation Solution at 37 °C for 5 minutes and then collected by trituration; Jurkat is a suspension cell line and was pipetted three times with a 10-ml serological pipette to generate single-cell suspension. After collection, cells were washed once with DPBS^−/−^ and counted (trypan blue staining, hemocytometer), and 1 × 10^6^ cells per sample were transferred into a 96-well plate. To assess sensitivity of LSMEM2 to common cell dissociation reagents, LSMEM2-expressing 293T cells were treated with Cell Dissociation Solution (4 ml) for 10 minutes at 37 °C or Accutase (2.5 ml) for 5 minutes at room temperature or Dispase (2.5 ml) for 10 minutes at 37 °C or TrypLE (2.5 ml) for 3 minutes at room temperature. hPSC were washed with DPBS−/− and treated with 1 ml of Accutase for 5 minutes at room temperature. hPSC-CMs were treated with 0.5 U ml^−1^ of Liberase-TH, 50 U ml^−1^ of DNase in 1 ml of RPMI followed by 1 ml of TrypLE for 5 minutes at 37 °C or 2 mg ml^−1^ of collagenase B, 50 U ml^−1^ of DNase in 1 ml of RPMI for 20 minutes at 37 °C followed by 1 ml of TrypLE for 5 minutes at 37 °C or 2 mg ml^−1^ of collagenase B, 50 U ml^−1^ of DNase in 1 ml of RPMI for 20 minutes at 37 °C followed by 1 ml of Accutase for 20 minutes at room temperature. For all live cell flow, 10,000 events were collected. Gating of cells was performed with SSC-A × FSC-A on an LSRII flow cytometer (BD Biosciences) or with SSC-H × FSC-H on an Attune Nxt flow cytometer (Thermo Fisher Scientific). Single cells were gated using sequential gates of FSC-W x FSC-H and then SSC-W × SSC-H. Live cells were gated using SSC-A × DAPI on LSRII or SSC-H × DAPI on Attune. FlowJo (version 10.7.2) was used to analyze flow cytometry data. Details for all antibody-based (antibodies, clones, amount, incubations times and buffers) assays are in Supplementary Table 15.

### RT–PCR

All surfaces, utensils and equipment were cleaned with RNAse-Away (Thermo Fisher Scientific, 7002). Tissues were processed using a Qiagen RNeasy Fibrous Mini Kit (74707) and homogenized using a bead disrupter (OMNI Bead Ruptor Elite with Cryo Unit; OMNI International, 19-620) kept on dry ice. RNA levels were quantified using a NanoDrop 2000. Then, 150 ng of RNA was converted to cDNA through a High-Capacity RNA-to-cDNA Kit from Invitrogen (4837406). Next, 10 ng of cDNA templates were combined with TaqMan Gene Expression Master Mix (Applied Biosystems, 4444557) and RNAse-free water. The reaction mixture was added to a 96-well PCR plate, followed by a specific TaqMan Assay (Hs00414922_g1 (*LSMEM2*), Hs99999901_s1 (*18S*)). Samples were analyzed using a Bio-Rad CFX Connect Real-Time PCR Detection System for 2 minutes at 50 °C, 10 minutes at 95 °C and 40 cycles of 15 seconds at 95 °C and 1 minute at 60 °C. Data were acquired using Bio-Rad CFX Maestro 4.1.2433.1219 and analyzed using GraphPad Prism 9.

### Isolation of primary cardiomyocytes from failing and non-failing cryopreserved heart tissue for targeted MS quantification of LSMEM2

Four samples each of failing and non-failing human cryopreserved left ventricular heart tissue were used for cardiomyocyte isolation using an improved cardiomyocyte isolation protocol where the Percoll gradient used above was replaced with laminar washing for improved efficiency and yield. Samples were sliced into 200-μm sections using a Leica VT1200S vibrating blade microtome and then digested in 300 U ml^−1^ of collagenase type 4, 0.1 mg ml^−1^ of trypsin inhibitor and 5 U ml^−1^ of DNase I using a 50-ml spinner flask at 37 °C. Crude cardiomyocytes were passed through a 200-μm strainer and collected by centrifugation at 80 *g* for 5 minutes at 4 °C with cells resuspended in unoxygenated ice-cold wash solution. To remove cellular debris, 50 μl of crude cardiomyocyte cell suspension was transferred into each well of a 96-well laminar flow plate and washed with unoxygenated ice-cold wash solution three times on a Laminar Wash HT2000 system (Curiox Biosystems) using the following parameters: flow rate 10 μl s^−1^, initial volume 80 μl and wash number 10. Purified cardiomyocytes were collected from each well and pooled.

### Isolation of primary cardiomyocytes from cryopreserved heart tissue and flow cytometry analysis of LSMEM2

Cardiomyocytes were isolated from human cryopreserved IVS section as described in the previous section using the laminar wash method. Cardiomyocytes were divided into two groups (*n* = 3): untreated and TrypLE treated. TrypLE treatment was carried out for 5 minutes, followed by centrifugation at 80 *g* for 5 minutes. Cell pellets were resuspended in 1 ml of HBSS^−/−^ containing 3% BSA. After 15 minutes of blocking at 4 °C, cells were washed using Laminar Wash HT2000 system. Each wash was carried out at 10 µl s^−1^ speed and ten rinses per cycle in HBSS. All methodological details (antibodies, clones, amount, incubations times and buffers) are described in Supplementary Table 15. Blocking was followed with antibody/isotype control incubation for 1 hour and a laminar wash to remove unbound antibody. Gating of cells was performed with SSC-H × FSC-H on an Attune Nxt flow cytometer (Thermo Fisher Scientific). Single cells were gated using sequential gates of FSC-W × FSC-H and then SSC-W × SSC-H. Live cells were gated using SSC-H × DAPI on Attune. FlowJo (version 10.7.2) was used to analyze flow cytometry data.

### Targeted MS data acquisition from cells and cardiac tissue

Cells or tissue for LSMEM2 targeted (SureQuant) assay were processed using an S-trap protocol as previously described^62^. Assays were applied to HeLa, Jurkat and U2-OS cells and isolated cardiomyocytes described above from four failing and four non-failing donors or whole LV tissue from four non-failing and six failing donors. To build an assay panel, unmodified, proteolytic and unique peptide precursors and fragments were identified for LSMEM2 in DDA mode. A survey panel was built using the default settings in SpectroDive version 11 (Biognosys). Stable isotopically labeled peptides were spiked into a 2-µg sample injection along with iRT synthetic peptides (Biognosys), and the resulting survey run was used in SpectroDive to build a SureQuant mass trigger list. For SureQuant experiments, a full scan was used to monitor for each heavy-labeled peptide and, when found above a default threshold, fragmented in low-resolution MS2 scan. The resulting fragments were used to trigger a second MS2 of the endogenous peptide, which elutes at the same time as the heavy peptide. All instrument and analysis settings are described in Supplementary Table 14.

### Reporting summary

Further information on research design is available in the Nature Portfolio Reporting Summary linked to this article.

### Supplementary information

Reporting Summary

Supplementary Table Identified proteins, peptides, donor information, reagent information and data acquisition settings.

### Source data

Source Data Fig. 4 Excel file with numerical raw data.

Source Data Fig. 7 Excel file with numerical raw data.

Source Data Fig. 8 Excel file with numerical raw data.

Source Data Extended Data Fig./Table 1 Excel file with numerical raw data.

Source Data Extended Data Fig./Tables 2 and 3 Excel file with numerical raw data.

Source Data Extended Data Fig./Table 5 Excel file with numerical raw data.

## Data Availability

Accession numbers for all proteins identified are available in the supplementary tables. Mass spectrometry raw files are available at MassIVE (https://massive.ucsd.edu/ProteoSAFe/static/massive.jsp) under accession number MSV000088624. All other data supporting the findings in this study are included in the manuscript and related files.

## Code availability

Veneer is freely available at https://www.cellsurfer.net/veneer, and code is available at https://github.com/GundryLab.
