# Supplementary material for: Surfaceome mapping of primary human heart cells with CellSurfer uncovers cardiomyocyte surface protein LSMEM2 and proteome dynamics in failing hearts
Source: Nat Cardiovasc Res. 2023 Jan 16;2(1):76–95. doi: 10.1038/s44161-022-00200-y (PMC10030153; doi:10.1038/s44161-022-00200-y)
Supplement: Supplementary file 1 — Reporting Summary [file 44161_2022_200_MOESM1_ESM.pdf]

nature portfolio

Corresponding author(s): Rebekah Gundry

Last updated by author(s): Nov 23, 2022

Reporting Summary

Nature Portfolio wishes to improve the reproducibility of the work that we publish. This form provides structure for consistency and transparency in reporting. For further information on Nature Portfolio policies, see our Editorial Policies and the Editorial Policy Checklist.

Statistics

For all statistical analyses, confirm that the following items are present in the figure legend, table legend, main text, or Methods section.

n/a	Confirmed	
☐	☒	The exact sample size (n) for each experimental group/condition, given as a discrete number and unit of measurement
☐	☒	A statement on whether measurements were taken from distinct samples or whether the same sample was measured repeatedly
☐	☒	The statistical test(s) used AND whether they are one- or two-sided<br>*Only common tests should be described solely by name; describe more complex techniques in the Methods section.*
☒	☐	A description of all covariates tested
☒	☐	A description of any assumptions or corrections, such as tests of normality and adjustment for multiple comparisons
☐	☒	A full description of the statistical parameters including central tendency (e.g. means) or other basic estimates (e.g. regression coefficient) AND variation (e.g. standard deviation) or associated estimates of uncertainty (e.g. confidence intervals)
☐	☒	For null hypothesis testing, the test statistic (e.g. F, t, r) with confidence intervals, effect sizes, degrees of freedom and P value noted<br>*Give P values as exact values whenever suitable.*
☒	☐	For Bayesian analysis, information on the choice of priors and Markov chain Monte Carlo settings
☒	☐	For hierarchical and complex designs, identification of the appropriate level for tests and full reporting of outcomes
☐	☒	Estimates of effect sizes (e.g. Cohen's d, Pearson's r), indicating how they were calculated

Our web collection on statistics for biologists contains articles on many of the points above.

Software and code

Policy information about availability of computer code

Data collection	Attune™ Cytomeric Software 5.1.2111.1; ZEN 2.3.69.1000; Thermo acquisition 3.1.231.6 and 4.1.261.18; Bio-Rad CFX Maestro 4.1.2433.1219;
Data analysis	MS data were processed with ProteomeDiscoverer 2.4 (Thermo Fisher Scientific), implementing Sequest HT and MSFragger 2.4 search algorithms followed by Percolator for post-search validation. DIA data were analyzed in Spectronaut™ 15 (Biognosys, Switzerland) using Pulsar™. FlowJo (version 10.7.2) was used to analyze flow cytometry data. ZEN imaging software 2.3.69.1000 and Image J were used to process images. Veneer is freely available at www.cellsurfer.net/veneer and code is available at https://github.com/GundryLab. GraphPad Prism 9.R (version 4.1.1), Python (version 3.6.9), UpsetR (https://github.com/hms-dbmi/UpSetR-shiny/) R package Pathfinder (https://cran.r-project.org/web/packages/pathfindR/index.html)

For manuscripts utilizing custom algorithms or software that are central to the research but not yet described in published literature, software must be made available to editors and reviewers. We strongly encourage code deposition in a community repository (e.g. GitHub). See the Nature Portfolio guidelines for submitting code & software for further information.

Data

Policy information about availability of data

All manuscripts must include a data availability statement. This statement should provide the following information, where applicable:

- Accession codes, unique identifiers, or web links for publicly available datasets
- A description of any restrictions on data availability
- For clinical datasets or third party data, please ensure that the statement adheres to our policy

Mass spectrometry raw files are available at MassIVE (massive.ucsd.edu) under the accession number MSV000088624.

Field-specific reporting

Please select the one below that is the best fit for your research. If you are not sure, read the appropriate sections before making your selection.

☒ Life sciences ☐ Behavioural & social sciences ☐ Ecological, evolutionary & environmental sciences

For a reference copy of the document with all sections, see nature.com/documents/nr-reporting-summary-flat.pdf

Life sciences study design

All studies must disclose on these points even when the disclosure is negative.

Sample size	Sample sizes were determined based on donor tissue availability. Due to limited availability of matched donors for quantitative cardiomyocyte and cardiac fibroblast uCSC experiments, 3 donors were chosen for each study (left and right ventricular tissue for cardiomyocyte study and apical tissue for cardiac fibroblast study). For quantitative uCSC cardiomyocyte analysis of LA and RA, 6 donors were pooled to obtain enough material for LC-MS/MS analysis. For qualitative uCSC studies, 9 donors were used. For quantitative uCSC of cardiomoycytes we used 3 non-failing and 4 failing donors. For qRT-PCR we used 8 non-failing and 6 failing donors; for targeted MS (SureQuant) of whole tissue we used 4 non-failing and 6 failing donors; for targeted MS (SureQuant) of isolated cardiomyocytes we used 4 donors each for failing and non-failing donors.
Data exclusions	No data were excluded
Replication	We first tested the performance and reproducibility of µCSC using RPMI 1788 and subsequently found it to be robust when applied to multiple different cell types (>100 experiments). We verified use of Ruby10.1 for tissue imaging and have performed >8 successful experiments using 4 different donors. We first verified use of Ruby10.1 antibody on HEK293T cells overexpressing LSMEM2 (3 experiments) and hPSC-CM then two other laboratories also successfully replicated results using different cell lines and differentiation methods (2 replicates each in 2 different laboratories for flow cytometry, a 3rd laboratory for imaging). All experiments contained negative and positive controls and were consistent among all 3 laboratories showing Ruby10.1 recognizes cardiomyocytes. Imaging of isolated cardiomyocytes showed similar results for cardiac actin (10 experiments) and streptavidin/biotin (5 experiments). No experiments included in this study had failed replication attempts that were not included.
Randomization	MS samples were randomized using randomizing function in Airtable. During MS data acquisition when comparing among chambers or disease vs control, the sample queue (order in which MS data are acquired) were blocked by technical injection and within each block the injection order was randomized. Pooled QC samples were acquired before, between, and after each sample block. This is not a clinical study so patients were not randomized. Rather, samples were allocated into the failing group if donor had been clinically diagnose with non-ischemic cardiomyopathy. Samples were allocated into the non-failing group if the donor had no clinical evidence of heart failure documented at time of autopsy.
Blinding	We did not use blinding as this is not a clinical trial or clinical study. No critical conclusions were drawn from any results where subjective classification or quantitation could have introduced observer bias.

Reporting for specific materials, systems and methods

We require information from authors about some types of materials, experimental systems and methods used in many studies. Here, indicate whether each material, system or method listed is relevant to your study. If you are not sure if a list item applies to your research, read the appropriate section before selecting a response.

Materials & experimental systems

n/a	Involved in the study	
☐	☒	Antibodies
☐	☒	Eukaryotic cell lines
☒	☐	Palaeontology and archaeology
☒	☐	Animals and other organisms
☐	☒	Human research participants
☒	☐	Clinical data
☒	☐	Dual use research of concern

Methods

n/a	Involved in the study	
☒	☐	ChIP-seq
☐	☒	Flow cytometry
☒	☐	MRI-based neuroimaging

Antibodies

Antibodies used	All details are provided in the supplement.<br>Item Vendor Catalog Clone name Lot number<br>Mouse Ruby 10.1 IgG1 Genscript Custom made 8D8A9-1 N/A<br>Mouse Ruby 10.1 IgG1 Alexa 647 Genscript Custom made 8D8A9-1 N/A<br>Mouse IgG1 isotype control, BD Biosciences, 557273<br>Mouse anti-human troponin I Fitzgerald 10R-T123k C5 4326

Anti-mouse IgG1 AlexaFluor 647, Life Technologies, A21240
Mouse IgG1 Isotype control AlexaFluor 647, R&D Systems, IC002R
Anti-mouse IgG1 AlexaFluor 488, Invitrogen, A21121
Mouse IgG2b isotype control, eBiosciences, 14-4732
Mouse anti-troponin I, US Biological T8665-13F
Rabbit anti-Perilipin, Abcam, Ab3526
Goat anti-mouse IgG2B AlexaFluor 594, Invitrogen A21145
Goat anti-mouse AlexaFluor 488, ThermoFisher A11029
Rabbit anti-goat AlexaFluor 568, ThermoFisher, A11079
Anti-human IgG1 cardiac actin Sigma-Aldrich A9357 AC1-20.4.2 075M4855V
Streptavidin FTIC BD Pharmingen 554060 N/A 8137939
Goat anti-mouse IgG1 AlexaFluor 568, Invitrogen A21124
Goat anti-rabbit IgG AlexaFluor 568, Invitrogen A11006
Goat anti-rabbit IgG AlexaFluor 488, Invitrogen A11008
Alexa Fluor 647 anti-vimentin Abcam Ab194719 EPR3776 GR3353000-5
Rabbit Anti-Vimentin IgG Cell Signaling Technologies (Danvers, MA) 5741S
Rabbit Anti-Cardiac Troponin I3 IgG Cell Signaling Technologies 13083 D6F8 1
Alexa Fluor 568 Rabbit anti goat A11079, ThermoFisher
Alexa Fluor 488 a11029, ThermoFisher
Anti-Kir2.1/KCNJ2 Almone Labs APC-026 N/A APC026AN1650
Anti-Nav1.5/SCN5A Almone Labs ASC-005 N/A ASC005AG1340
Anti-Connexin 43 Cell Signaling Technology 3512S N/A

Validation

All antibodies were titrated and tested using positive and negative controls, including secondary only and antigen blocking.
Ruby10.1 antibody was validated for flow cytometry using HEK293T (negative control) and HEK293T-overexpressing LSMEM2 (positive control). Blocking peptides were applied to positive control to ensure signal was specific.
Ruby10.1 was validated for immunofluorescence imaging using human heart tissue. Blocking peptides, secondary-only controls were applied to ensure signal was specific. Co-stains of Ruby10.1 with vimentin for non-cardiomyocytes, and troponin, KCNJ2, SCN5A for cardiomyocytes shows that Ruby10.1 only recognizes cardiomyocytes in tissue. In hPSC-CM immunofluorescence imaging, Ruby10.1 was co-stained with troponin and confirmed to recognized cardiomyocytes.
Cardiac actin antibody was previously validated in isolated cardiomyocytes (PMID: 34437879).
KCNJ2, SCN5A, Troponin, CX43 and Vimentin antibodies were previously validated for immunofluorescence imaging of tissue. Here, Vimentin was used in cardiac tissue and was specific to endothelial cells and cardiac fibroblasts. Antibodies to KCNJ2, SCN5A, Troponin and Vimentin data are consistent with vendor datasheets and published literature regarding cell type specificity and subcellular location. Anti-Kir2.1/KCNJ2 Ab has been used in >100 citations, and stains myocytes (example Melnyk, P. et al. (2002) Am. J. Physiol. 283, 1123.) and Anti-SCN5A Ab has been used in >150 citations, and stains myocytes (example Casini et al. Cardiovasc Research, 85, 691; 2010). Anti-Troponin Ab performs as expected results and vendor data as the protein is unique to cardiomyocytes. Anti-CX43 has been used in >200 citations and stains intercalated discs in cardiac tissue (example Kells-Andrews et al., J Cell Si, 131(15), 2018). Anti-Vimentin Ab has been used in >2000 citations and stains many different cell types, including fibroblasts (example Stancil et al., Nat Comm, 2021).

Eukaryotic cell lines

Policy information about cell lines

Cell line source(s)

ITEM VENDOR CATALOG
DF6-9-9T WiCell iPS DF6-9-9T.B
JHU001, created by collaborator
H7, WiCell
RPMI 1788 ATCC CCL-156
HeLa ATCC CCL-2
U-2 OS ATCC HTB-96
Jurkat, clone E6-1 ATCC TIB-152
293T ATCC CRL3216
PRIMARY - not an immortalized line: Primary coronary artery smooth muscle cells (HCASMC) ATCC PCS-100-021
RRIMARY - not an immortalized line: Human cardiac microvascular endothelial cells Lonza CC-7030

Authentication

All purchased cell lines were shipped with authentication certificate from ATCC which uses short tandem repeat profiling to authenticate identity. ATCC provided PDF Certificate of Analyses for each lines with testing dates of 6/8/2019 (U2OS); 9/22/2020 (HeLa); 2/5/2009 (RPMI1788); and 12/27/2019 (Jurkat). These testing dates are same or close to when vials were frozen by the vendor. We thawed and passaged minimally prior to use for end assays. To authenticate the hiPSC lines used in this study, we adhere to the recommendations of Maherali and Hochedlinger. The lines were confirmed to self-renew, have appropriate morphological attributes (e.g., high nuclear to cytoplasmic ratio, colony formation), exhibit appropriate cell surface markers (e.g., SSEA4, Tra-1-60/1-81) by flow cytometry, express key pluripotency gene transcripts and proteins (e.g., Oct4, Sox2, Nanog) by qPCR and immunostaining, respectively without expression of lineage-specific genes associated with the cell of origin. All lines have been karyotyped (by ThermoFisher) and were determined to be normal. Upon differentiation, the cells were found to have a normal morphology, contract spontaneously, were selected with lactate medium and found to be cardiac troponin T positive (at ~90-95%) by flow cytometry. Cells that were less than 85% cTnT positive were excluded from the analyses.

Mycoplasma contamination

All cell lines were testing monthly for mycoplasma contamination using the MycoAlert PLUS Mycoplasma Detection Kit from Lonza (catalog number LT07-710). All cell lines were below a ratio of 1, which indicates that cell lines were not contaminated with mycoplasma.

Commonly misidentified lines (See ICLAC register)	We did not use any commonly misidentified cell lines.

Human research participants

Policy information about studies involving human research participants

Population characteristics	Adults (18 yo or older) who are undergoing heart transplantation or implantation of a left ventricular assist device or rapid autopsy. No exclusions based on race, sex. Any infectious disease are excluded. Full details of donors used for each set of experiments are described in detail in Extended Data Tables 11 and 12.
Recruitment	All specimens used in this study are obtained as deidentified samples from institutional biobanks where specimens are collected from consented donors under IRB-approved studies. Patients undergoing heart transplant or implantation of left ventricular assist device are approached by the clinical provider to ask if they are interested in donating discarded tissue removed during surgery to the biobank. Informed consent is then obtained from any participant who decides to participate. Specimens are collected by the biobank. The biobank then provides these specimens as deidentified samples to the researcher. Donors do not receive any compensation for participating. Family provides consent for rapid autopsy donors.
Ethics oversight	Medical College of Wisconsin and University of Nebraska Medical Center provided ethical oversight and approval of IRB for the study. Materials were obtained as deidentified material from the institutional tissue banks under institutional review board approvals (PRO00025506; PRO643-17-EP).

Note that full information on the approval of the study protocol must also be provided in the manuscript.

Flow Cytometry

Plots

Confirm that:

☒ The axis labels state the marker and fluorochrome used (e.g. CD4-FITC).

☒ The axis scales are clearly visible. Include numbers along axes only for bottom left plot of group (a 'group' is an analysis of identical markers).

☒ All plots are contour plots with outliers or pseudocolor plots.

☒ A numerical value for number of cells or percentage (with statistics) is provided.

Methodology

Sample preparation	All details are provided in supplementary information.<br>Preparation of single cell suspensions<br>To generate single cell suspensions for 293T, HeLa, U2-OS, and Jurkat cell lines, cells were washed once with DPBS-/- and dissociated using cell-type specific conditions: For Ruby10.1 immunodetection, 293T were washed twice in DPBS-/- and then incubated in 0.5 mM EDTA in DPBS-/- at 37°C for 10 min. EDTA solution was removed and cells were further incubated in DPBS-/- for 10 min at room temperature before collection by trituration. 293T, HeLa, and U2-OS were washed twice in DPBS-/- and then incubated in cell dissociation solution at 37°C for 5 min then collected by trituration; Jurkat is a suspension cell line and was pipetted three times with 10 mL serological pipet to generate single cell suspension. Following collection, cells were washed once with DPBS-/-, counted (trypan blue staining, hemocytometer), and 1x106 cells per sample were transferred into a 96-well plate.<br><br>Live cell flow cytometry for LSMEM2 staining<br>All steps were performed on ice or at 4°C. Cells were blocked with 3% BSA in HBSS-/- for 15 min. Antibody details are provided below. Primary antibodies were added to cells and gently agitated on orbital shaker for 45 min. Antibody details can be found in Table S2 and Table S3. Cells were washed three times with 3 mL of HBSS-/-. Secondary antibodies and DAPI were pre-diluted in block, used to resuspend cells post-wash, and then gently agitated on orbital shaker for 30 min. Cells were washed three times with 3 mL of HBSS and then resuspended in 0.3 mL of block and transferred to filter-cap flow tubes for analysis by flow cytometer. For all live-cell flow, 10,000 events were collected. Gating of cells was performed with SSC-A x FSC-A on a LSRII flow cytometer (BD Biosciences) or with SSC-H x FSC-H on an Attune Nxt flow cytometer (Thermo Fisher Scientific). Single cells were gated using sequential gates of FSC-W x FSC-H and then SSC-W x SSC-H. Live cell were gated using SSC-A x DAPI on LSRII or SSC-H x DAPI on Attune. FlowJo (version 10.7.2) was used to analyze flow cytometry data.
Instrument	For all live-cell flow at least 10,000 events were collected. Gating of cells was performed with SSC-A x FSC-A on LSRII flow cytometer (BD Biosciences) or with SSC-H x FSC-H on Attune Nxt flow cytometer (Thermo Fisher Scientific).
Software	FlowJo version 10.7.2 or 10.8.1
Cell population abundance	No sorting experiments were performed.
Gating strategy	Live: Single cells were gated using sequential gates of FSC-W x FSC-H and then SSC-W x SSC-H. Doublets were excluded using SSC-A x SSC-H and FSC-A x FSC-H or SSC-W x SSC-H and FSC-W x FSC-H. Live cell were gated using SSC-A x DAPI on LSRII or SSC-H x DAPI on Attune. Gating example is shown in Extended Data Fig. 6a.

☒ Tick this box to confirm that a figure exemplifying the gating strategy is provided in the Supplementary Information.
